# Risk-based sensitivity analysis of urban gas infrastructure in new district planning

**DOI:** 10.1038/s41598-026-51283-7

**Published:** 2026-05-25

**Authors:** Lei Gao, Hao Liu, Hui Lan, Zhenyu Zhao, Cui Li, Ting Zhu

**Affiliations:** 1 School of Economics and Management, University of Emergency Management, Sanhe, 065201 China; 2 Department of Disciplines and Graduate Studies, University of Emergency Management, Sanhe, 065201 China; 3 Hubei Key Laboratory of Blasting Engineering, Jianghan University, Wuhan, 430056 China; 4 School of Artificial Intelligence, Jianghan University, Wuhan, 430056 China; 5 School of Economics and Management, North China Electric Power University, Beijing, 102206 China

**Keywords:** Gas infrastructure resilience, System dynamics-based framework, Risk sensitivity analysis, Urban planning, Engineering, Environmental sciences, Environmental studies, Natural hazards

## Abstract

Urban gas infrastructure resilience is critical for public safety, yet rapidly expanding networks face increasing vulnerability. This study develops a system dynamics-based framework to identify key leverage points for enhancing gas infrastructure resilience in new urban districts. Using a three-tier indicator system of 32 risk factors across four dimensions, analytic hierarchy process weighting is integrated with system dynamics modeling in a case study of a new district in China. Sensitivity analysis reveals that system’s inherent resilience contributes most significantly, with the proportion of high-pressure pipelines exhibiting the highest sensitivity, followed by population-infrastructure spatial coupling and building seismic resistance. These findings demonstrate that planning-phase interventions targeting high-sensitivity indicators offer the most efficient pathway to enhancing urban gas infrastructure resilience, challenging conventional priorities that emphasize emergency response over foundational design considerations.

## Introduction

Urbanization increases reliance on critical infrastructure like gas networks, essential for public safety, property protection, and sustainable development^1^. In China, rapid urbanization drives significant natural gas demand and infrastructure expansion^2,3^, supported by national energy strategies aiming to increase gas consumption share and strengthen infrastructure^4–7^. However, regional sustainability disparities persist^8–10^.

Gas system safety is critical due to its high societal impact^11^. Supporting this, Lei Pang’s analysis of 6,063 accidents (2012–2021) found 81% occurred in residential, construction, and commercial areas^12,13^. Research addresses vulnerabilities from macro-planning, e.g., pipeline risk assessment frameworks and land use advice^14–16^, and micro-safety perspectives, e.g., safety distances for gas releases^17^. Therefore, pipeline vulnerability studies examine physical performance, design, operation, maintenance, environmental effects, and emergency response^18,19^, including corrosion assessed by Bayesian networks^20–22^ and third-party damage by Bayesian theory and fault trees^23,24^. In addition, system improvements involve safety-oriented operation algorithms^25^. Emergency response research covers efficiency models^26^, compliance frameworks^27^, evacuation models^28,29^,and road optimization to reduce the impact of disasters^30,31^.Gas leak and transport studies explore explosion patterns^32^, detection technologies^33–35^, fire impacts^36^, hydrate prediction^37^, and accident analysis^38^.

As critical infrastructure, gas systems exhibit significant vulnerability to extreme weather and natural disasters, potentially causing leaks, fires, explosions^39^, cascading failures like widespread service interruptions^40,41^, and major urban disruptions^41^. Therefore, disaster risk management is vital for urban planning, requiring comprehensive risk assessment, prevention, response, and community engagement. Urban gas accidents unfold as continuous processes with variable durations^42^, necessitating planning that extends beyond emergency response to source risk management. Strategies include environment redesign for disaster prevention^43^, participatory risk reduction^44^, enhancing local government efforts^45^, and assessing local capabilities^46^. However, gaps exist in current disaster planning. This is mainly reflected in the fact that planning often overlooks local hazards and vulnerabilities^47^, lacks fundamental understanding, clear phase-specific actions, resident involvement^48^, and lack of indicators to monitor disaster progression^49^. In this context, integrating science and technology is recognized as key to improving disaster management^50^. Relevant studies explore the potential and limitations of application of ChatGPT in disaster^51^, future complex disaster trends and safety technologies^52^, highlighting the integration of new technologies and interdisciplinary approaches as active research frontiers.

In summary, gas infrastructure is vital for urban safety and sustainability, especially in rapidly urbanizing China. Despite its essential role, these systems face significant vulnerabilities to both accidents and disasters, driving extensive research into risk assessment, pipeline safety, and emergency response. While current disaster planning emphasizes comprehensive risk management, it suffers from critical gaps, including inadequate local hazard consideration and unclear actionable strategies. To address these identified needs, this study develops a risk indicator system and a system dynamics-based framework specifically for new urban areas, aiming to identify key vulnerabilities and ultimately enhance the resilience of gas infrastructure. It does these by investigating which components of an urban gas system’s risk and resilience architecture yield the greatest marginal improvements in integrated resilience when adjusted at the planning stage. While previous studies have applied system dynamics to operational pipeline safety or used AHP for risk weighting, our framework uniquely integrates these methods within a planning-stage context and explicitly links indicator sensitivity to actionable design decisions, quantifying the relative marginal benefits of planning-phase interventions.

## Methods

This study employs a mixed-methods research design. First, an indicator-based risk assessment system is constructed drawing from literature, policy documents, and semi-structured expert interviews. Second, the Analytic Hierarchy Process (AHP) is applied to derive indicator weights. Third, these indicators are embedded in a system dynamics model implemented in Vensim to simulate resilience trajectories and conduct sensitivity analysis. This approach was chosen over purely empirical models due to data limitations in new districts, and over agent-based models as the focus is on system-level feedback. The combination of expert knowledge with dynamic simulation allows us to capture complex interdependencies.

### Indicator system

A multi-facet qualitative approach is adopted in this study to identify a list of factors critical for gas infrastructure risks. The study unfolds in a combination of literature survey, review of policy documents and interviews.

(1) Literature survey.

An extensive review of relevant literature was conducted, including the following two sources.

Research Papers: A systematic search was carried out in major databases using keywords such as “gas system safety,” “pipeline safety,” “gas risk,” and “gas accident” to find relevant papers published from 2000 to 2024. Chinese academic databases were also searched for local publications on gas safety.

Statistical Data: Statistical data were retrieved from the China Urban Construction Statistical Yearbook. Data on the total gas supply, pipeline lengths, supply capacity, and gas-consuming population for 2021 were compiled. This data includes information on gas supply, pipeline lengths, supply capacity, and gas-consuming populations.

(2) Review of policies.

Policy documents were sourced from various official publications and regulations released by major government authorities, including the China Urban Management and Law Enforcement, Municipal Government, County Government, District Government, Bureau of Natural Resources and Planning, Municipal Housing and Urban-rural Development Bureau, Provincial Department of Housing and Urban-Rural Development, County Housing and Urban-rural Development Bureau.

(3) Semi-structured interviews.

**Fig. 1:**
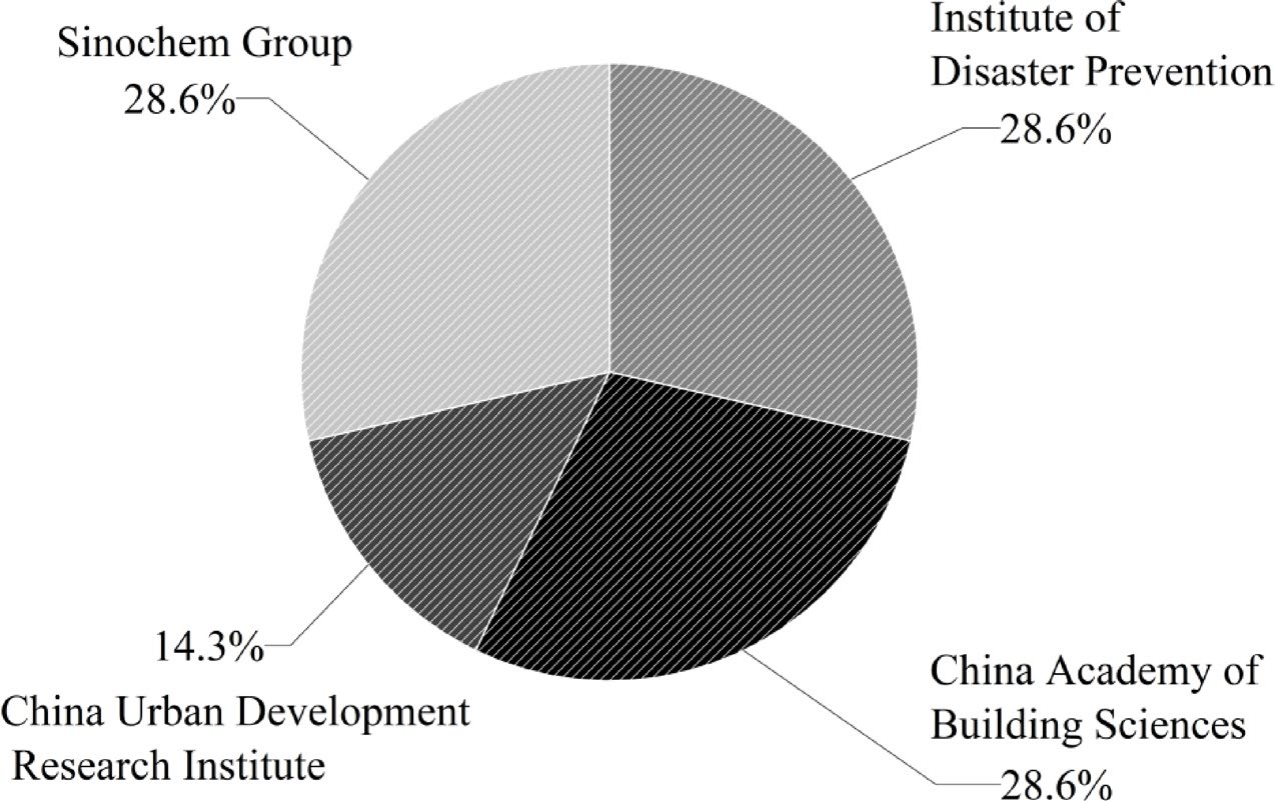
The distribution of interviewees’ work organizations.

The objective of the interviews is to score the indicators and assess the risk of the gas system. A total of seven semi-structured interviews were conducted with selected practitioners involved in the Chinese gas industry, and the categories of interviewees are shown in Fig. 1. While the sample size of seven experts may appear limited, it is appropriate given their deep domain expertise, with an average of 15 years of experience. To mitigate bias, several strategies were employed: experts were drawn from diverse backgrounds; a Delphi-style process allowed them to revise scores after reviewing anonymized group feedback; all pairwise comparison matrices satisfied the consistency ratio (CR < 0.1); and inter-rater reliability was assessed using Kendall’s coefficient of concordance (W = 0.72, *p* < 0.01), indicating substantial agreement beyond chance.

All methods involving human participants were carried out in accordance with relevant guidelines and regulations. Informed consent was obtained from all expert participants prior to the interviews. As the study involved only professional expert elicitation and did not include any experimental intervention or collection of personal health data, formal ethics committee approval was not required according to institutional guidelines.

Finally, an indicator system was established. An initial pool of 47 candidate indicators was identified through literature and policy review. Through expert interviews and two rounds of Delphi-style feedback, 15 indicators were excluded due to redundancy, lack of planning-stage relevance, or measurement difficulty. The final set of 32 represents those judged by expert consensus as both relevant and amenable to assessment. The hierarchical structure model is established. The highest level is the first level risk factors of the gas system, and then the 2 and 3 indicator levels are established in turn. As it is shown in Table 1.

The following definitions were adopted: risk refers to the combination of hazard probability and consequence; vulnerability describes the susceptibility to damage; fragility refers to the conditional probability of failure; and resilience encompasses the capacity to anticipate, absorb, and recover from hazardous events.

**Table 1:** Risk assessment indicator system of regional gas infrastructure.

Level-1 indicators	Level-2 indicators	Level-3 indicators
Natural disaster risk prevention capability of gas system (A1)	Seismic engineering planning (B1)	Building seismic resistance (C1)
		Lifeline seismic safety (C2)
		Seismic secondary hazard prevention (C3)
	Flood control and drainage engineering planning (B2)	Flood control standards (C4)
		Flood engineering measures (C5)
		Flood non-engineering measures (C6)
	Geological disaster prevention and control planning (B3)	Geohazard assessment (C7)
		Geohazard mitigation measures (C8)
Gas System’s inherent resilience (A2)	Assessment of potential hazard sources (B4)	LNG station siting (C9)
		CNG fueling station siting (C10)
		Major hazard protection (C11)
	Pipeline facility safety plan (B5)	Proportion of high-pressure and Sub-HP pipelines (C12)
		Permanent markings (C13)
	Third party pipeline damage and on-site protection planning (B6)	Construction and pipeline signage (C14)
		On-site monitoring (C15)
	Pipeline corrosion risk protection (B7)	Pipeline corrosion factors assessment (C16)
		Pipeline corrosion protection (C17)
Resilience of gas surrounding environment (A3)	Resilience of surrounding population distribution (B8)	Population density (C18)
		Population-gas system spatial correlation (C19)
	Resilience of buildings surrounding gas systems (B9)	Building type resilience (C20)
		Building-gas system spatial correlation (C21)
	Resilience of surrounding infrastructure of gas system (B10)	Infrastructure distribution resilience (C22)
		Infrastructure-gas system spatial correlation (C23)
Emergency, rescue, and evacuation capabilities (A4)	Spatial correlation between land use and fire rescue facilities (B11)	Fire stations distribution (C24)
		Fire water supply (C25)
	Emergency support infrastructure planning (B12)	Emergency infrastructure level (C26)
		Lifeline response capability (C27)
	Emergency access and evacuation exit planning (B13)	Passage and exit distribution (C28)
		Passage and exit accessibility (C29)
	Emergency rescue planning (B14)	Emergency response teams (C30)
		Emergency communications and commands (C31)
		Emergency medical facilities (C32)

LNG: Liquefied natural gas stations; CNG: Compressed natural gas fueling stations; HP: High-pressure pipelines.

### Indicator weight analysis and consistency testing

#### Structure of comparison matrix

In order to determine the weights of different indicators in the evaluation index system, it is necessary to assign values to the weights of indicators at each level. Therefore, a judgment matrix needs to be created. The judgment matrix is a representation of comparing the importance of elements on the same hierarchy level with regards to a certain criterion in the level above. As it is shown in Table 2, the relative importance of indicator $\:{a}_{i}$ to indicator $\:{a}_{j}\:$can be denoted as $\:{a}_{ij}$. It’s important to note the scale of significance and meaning in Table 3, where numbers from 1 to 9 represent the relative importance of each hierarchical factor.

The judgment matrix should satisfy the Eq. (1).

1 $$\:\begin{array}{c}{a}_{ij}=\raisebox{1ex}{$1$}\!\left/\:\!\raisebox{-1ex}{${a}_{ij}$}\right.>0,{a}_{ii}=1\end{array}$$

a_ij_ and a_ji_ represent the relative importance between the two indicators.

**Table 2:** Criterion layer judgment matrix.

A	a_1_	a_2_	…	a_*n*_
a_1_	a_11_	a_12_	…	a_1n_
…	…	…	…	…
a_n_	a_n1_	a_n2_	…	a_nn_

**Table 3:** Importance rating and its meaning.

Importance rating	Meaning
1	The two factors are considered equally important.
3	The former is slightly more important than the latter of the two factors.
5	The former is clearly more important than the latter of the two factors.
7	The former is strongly more important than the latter of the two factors.
9	The former is exceptionally more important than the latter of the two factors.
2, 4, 6, 8	The intermediate values of the above degrees of judgments.

#### Weight analysis and consistency calculation

In this paper, the arithmetic average method is used to calculate the weights of each layer of indicators. The elements in the matrix are normalized by columns, and then the maximum eigenvalue $\:{{\uplambda\:}}_{\mathrm{m}\mathrm{a}\mathrm{x}}$ corresponding to the judgment matrix and its corresponding eigenvector w are calculated according to Eq. (2), Eq. (3) and Eq. (4).

2 $$\:\begin{array}{c}{{\Omega\:}}_{\mathrm{i}}=\frac{1}{\mathrm{n}}\sum\:_{\mathrm{j}=1}^{\mathrm{n}}\frac{{\mathrm{a}}_{\mathrm{i}\mathrm{j}}}{\sum\:_{k=1}^{n}{\mathrm{a}}_{\mathrm{k}\mathrm{j}}}\left(\mathrm{I},\:\mathrm{j}=\:1,\:2,\:\dots\:,\:\mathrm{n}\right)\:\end{array}$$

3 $$\:\begin{array}{c}Aw={{\uplambda\:}}_{\mathrm{m}\mathrm{a}\mathrm{x}}w\end{array}$$

4 $$\:\begin{array}{c}{{\Lambda\:}}_{\mathrm{m}\mathrm{a}\mathrm{x}}=\sum\:_{i=1}^{n}\frac{{\left[Aw\right]}_{i}}{n{w}_{i}}\:\end{array}$$

After calculating the weights of each matrix, it is necessary to carry out a one-time test on the discriminant matrix to check whether it meets the consistency requirements. First of all, the consistency index CI is calculated, and the formula is shown in Eq. (5).

5 $$\:\begin{array}{c}CI=\frac{{{\uplambda\:}}_{\mathrm{m}\mathrm{a}\mathrm{x}}-n}{n-1}\end{array}$$

Where: $\:{{\uplambda\:}}_{\mathrm{m}\mathrm{a}\mathrm{x}}\:$is the maximum eigenvalue of the judgment matrix. n is the order of the matrix.

Individual expert judgment matrices were aggregated using the geometric mean method, which preserves the reciprocal property of the matrices. All experts were weighted equally, as preliminary analysis showed no systematic differences based on their affiliations or experience that would justify differential weighting. The consistency ratio of the aggregated matrix is below the 0.1 threshold, indicating acceptable consistency. Then, the table is checked to get the average random consistency index (RI) corresponding to the judgment matrix, and the list of standard values of the average random consistency indicators are shown in Table 4.

**Table 4:** List of standard values for average random consistency indicators.

*n*	1	2	3	4	5	6	7	8	9	10	11
RI	0	0	0.52	0.89	1.12	1.26	1.36	1.41	1.45	1.49	1.51

Finally, the consistency ratio CR is calculated based on CI and RI, as shown in Eq. (6).

6 $$\:\begin{array}{c}CR=\frac{CI}{RI}\:\end{array}$$

When CR < 0.1, it means that the judgment matrix has consistency; when CR > 0.1, the judgment matrix needs to be reconstructed.

### System dynamics-based framework for gas infrastructure resilience

#### Model framework

Nature of the Modeling Approach. It is essential to clarify the specific application of the System Dynamics (SD) method in this study. Unlike traditional SD models that simulate endogenous feedback behavior (e.g., population growth stressing infrastructure, which then limits growth), the presented framework is a “static-dynamic hybrid.” The relationships among risk indicators are static and defined by AHP-derived linear weights. The core risk indicators are treated as parameters representing planning-stage design decisions rather than variables that evolve through internal system feedback. The “dynamic” aspect is limited to the time-phased implementation of planning interventions: over the 100-month simulation horizon, the model accumulates resilience improvements as infrastructure upgrades and policy changes are progressively rolled out. This approach does not capture emergent system behavior or feedback loops. Consequently, this work is more accurately described as a Multi-Criteria Sensitivity Analysis scaffolded on a System Dynamics architecture. The SD stock-flow structure is retained because it ensures internal mathematical consistency across the four-level indicator hierarchy and provides a transparent mechanism for visualizing the temporal accumulation of resilience gains, which purely static Multi-Criteria Decision Analysis (MCDA) methods cannot easily accommodate.

The modeling process for simulating gas infrastructure resilience involves the following key steps:

(1) Modeling objectives: The primary objective is to utilize SD sensitivity analysis to assess the impact of various indicators on system resilience. Prior to model development, a thorough analysis of the real problem is essential to clearly define the research scope and ensure SD methodology is appropriate for addressing the identified systemic issues.

(2) System boundaries: System boundaries are conceptual delineations established by focusing on the core problem. Factors exerting an essential influence on gas infrastructure resilience are included within the boundary, while factors with negligible impact are excluded. Defining modeling objectives guides the identification of relevant system boundaries.

(3) System structure: This system comprises interconnected subsystems. Based on identified causal relationships, a causal loop diagram (CLD) depicting the interactions among gas infrastructure risk factors is developed. Following boundary definition, key system variables and constants are identified, along with their types and attributes. The relationships between variables, constants and subsystems are analyzed to construct a comprehensive SD stock-and-flow diagram for gas infrastructure risk factors.

(4) Model formulation: The inherent dynamic linkages and feedback mechanisms within gas infrastructure risk factors necessitate expressing complex, non-linear relationships through mathematical equations. These equations are iteratively refined and simulated to develop a robust SD model.

(5) Model validation and application: The initial model undergoes rigorous validation to identify logical inconsistencies, requiring adjustments to structure, parameters, feedback mechanisms, or equations. Once validated as reasonable, the model is simulated using historical parameters. Comparison of simulation outputs with historical data reveals model deficiencies, guiding further refinement. Final adjustments are made through sensitivity and simulation testing until results align realistically with observed behavior. Model validation employed multiple approaches appropriate for planning-stage models, including structural validation, where causal relationships were reviewed by three external domain experts; parameter validation, where all values were checked against published standards where available; behavior pattern validation, where the model reproduces the expected pattern that inherent resilience dominates overall performance; and extreme condition testing, where the model was run with all indicators at minimum (0) and maximum (1), producing plausible resilience ranges.

(6) Simulation and evaluation: The validated model is employed within SD software to simulate system behavior under varying parameter conditions. The resulting outputs inform the development of scientifically strategies and policy recommendations.

#### Causal loop diagram (CLD)

The CLD was constructed iteratively. First, a preliminary diagram was drafted based on causal relationships identified in the literature. Second, this draft was presented to three experts for verification and refinement. Third, the revised diagram was reviewed by all seven experts in a workshop format, with consensus reached on the final structure. Causal links were considered ‘supported’ if confirmed by at least two literature sources or two experts.

Enhancing gas system resilience against natural disasters involves multiple interdependent factors. The characteristics of three important factors are as follows.

(1) Seismic resilience: Building seismic resistance and secondary disaster prevention directly protect gas infrastructure. Lifeline engineering seismic safety ensures critical infrastructure integrity.

(2) Flood resilience: Engineering and non-engineering flood control measures, coupled with elevated flood defense standards, reduce regional vulnerability and directly safeguard gas systems.

(3) Geohazard mitigation: Protective measures informed by geohazard risk assessments minimize geological threats to gas infrastructure.

Collectively, these aspects strengthen natural disaster defense capabilities.

Similarly, gas system’s inherent resilience involves multiple factors. The characteristics of two important factors are as follows.

(1) Facility siting: Optimal siting of LNG vaporization stations and CNG refueling stations reduces major hazard potential. Effective security measures further mitigate risk.

(2) Pipeline protection status: Managing the proportion of high-pressure and sub-high-pressure pipelines, implementing external corrosion protection, and enforcing on-site monitoring, patrols, and signage through comprehensive protection planning ensure holistic pipeline safety.

The resilience of the gas system’s surroundings is shaped by spatial interactions. The characteristics of three important factors are as follows.

(1) Resilience of surrounding population distribution: Determined by population density and its spatial distribution relative to gas infrastructure.

(2) Infrastructure resilience: Influenced by the proportion of high-pressure and sub-high-pressure pipelines. Similarly, determined by the spatial correlation between other infrastructure and gas system.

(3) Structural resilience: Defined by building characteristics and their spatial correlation with gas system.

These resilience dimensions collectively measure environmental vulnerability during gas incidents, crucial for integrated risk assessment.

Emergency response, rescue, and evacuation capabilities depend on synergistic factors. The characteristics of three important factors are as follows.

(1) Lifeline emergency capacity: Supported by fire stations, emergency infrastructure, water sources, contingency supplies, and critical facility security.

(2) Evacuation corridor capacity: Determined by corridor characteristics and regional disaster impact, enabling rapid evacuation.

(3) Search & rescue capacity: Built on emergency response teams, medical facilities, communication systems and command systems.

**Fig. 2:**
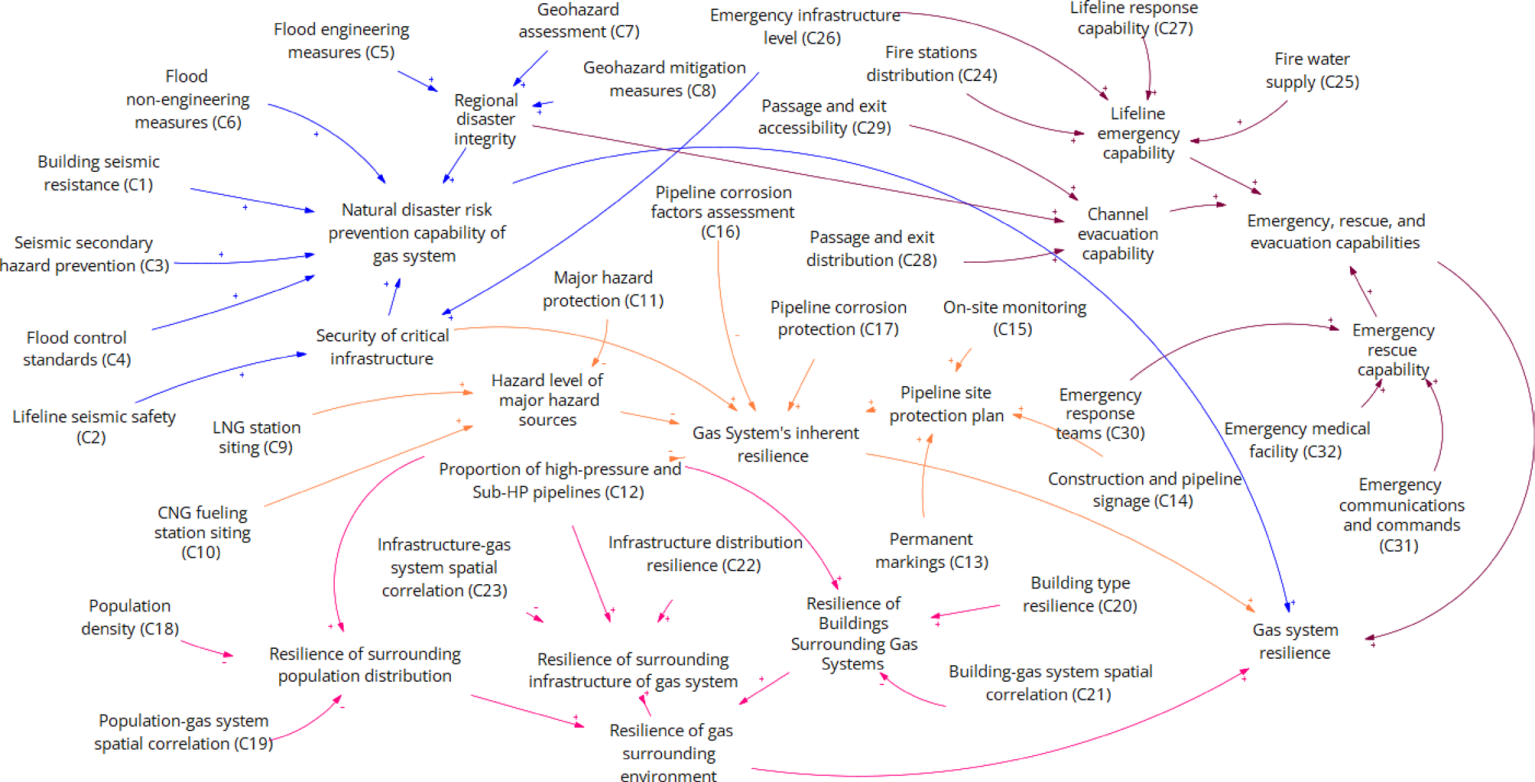
Causal Loop Diagram of risk factors for gas infrastructures.

In summary, these integrated capabilities form an efficient emergency management system. Subsequently, these factors including natural disaster resilience, gas system’s inherent resilience, resilience of gas surrounding environment and emergency, rescue, and evacuation capabilities were integrated. A unified CLD is constructed in Fig. 2, capturing the complex interdependencies within the urban gas system. In the CLD, arrows represent causal influences through specific mechanisms. For instance, the arrow from ‘Proportion of high-pressure pipelines’ to ‘Pipeline leak probability’ reflects that higher pressure leads to greater stress and higher failure rates. The arrow from ‘Population density’ to ‘Consequence severity’ captures that leaks in dense areas affect more people. The arrow from ‘Emergency response capability’ to ‘Consequence severity’ represents that faster response reduces exposure.

#### Parameter determination

Establishing parameter values for variables is critical in SD-based framework formulation. Based on relevant standards, data, and system stability considerations, parameters are determined by three primary methods: The first is for variables with known stable relationships, existing models provide reference values derived from expert experience, trend extrapolation, or arithmetic averaging. The second approach is to use traditional mathematical functions to model variables with clear and simple interdependencies. The third is to use table functions to represent nonlinear relationships involving incomplete data or fuzzy correlations.

Qualitative variables were operationalized through expert scoring on a 1–5 Likert scale, with detailed anchor descriptions. For example, for ‘building seismic resistance’, a score of 1 indicated ‘no seismic design’, while 5 indicated ‘advanced seismic design with base isolation’. These scores were then normalized to a 0–1 scale to serve as inputs.

Within this model, parameters were determined as follows:

(1) Initial value setting: Given the qualitative nature of most variables, data were obtained through expert scoring. Scores derived from a prior fuzzy comprehensive evaluation stage were normalized. All variables range from 0 (most hazardous state) to 1 (safest state).

(2) Factor weighting: Indicator weights were primarily determined using the Analytic Hierarchy Process (AHP). The decision problem was decomposed into a hierarchical structure (goal, sub-goals, criteria, alternatives). Weights for elements at each level relative to the level above were calculated by solving the eigenvector of pairwise comparison matrices. Final weights for alternatives relative to the integrated goal were obtained through weighted summation.

#### System equation formulation

Through iterative simulation and refinement, guided by established SD theory and the defined set of resilience-influencing variables, system dynamics equations were developed. These equations quantitatively represent the coupling relationships among the various influencing factors within the urban planning context. Functional forms were selected based on two principles: linear additive forms were used where variables contribute independently; expert judgment informed choices where theory was ambiguous, as shown in Table 5.

**Table 5:** Variables and expressions of system dynamics equations.

Variables	Types	Expressions
Gas system resilience	State	Gas system resilience =INTEG (Resilience changes of gas system)
Natural disaster risk prevention capability of gas system	State	Natural disaster risk prevention capability of gas system =INTEG (Change in Natural disaster risk prevention capability)
Gas system’s inherent resilience	State	Gas system’s inherent resilience =INTEG (Change in inherent resilience of gas system)
Resilience of gas surrounding environment	State	Resilience of gas surrounding environment =INTEG (Resilience variation of gas system)
Emergency, rescue, and evacuation capabilities	State	Emergency, rescue, and evacuation capabilities =INTEG (Changes in emergency, rescue, and evacuation capabilities)
Resilience changes of gas system	Rate	Resilience changes of gas system = Change in Natural disaster risk prevention capability + Change in inherent resilience of gas system + Resilience variation of gas system +Changes in emergency, rescue, and evacuation capabilities
Change in Natural disaster risk prevention capability	Rate	Change in Natural disaster risk prevention capability = Building seismic resistance$\:\times\:$weight$\:\times\:$proportion+ Regional disaster integrity$\:\times\:$weight$\:\times\:$proportion+ Security of critical infrastructure$\:\times\:$weight$\:\times\:$proportion+ Seismic secondary hazard prevention$\:\times\:$weight$\:\times\:$proportion+ Flood control standards$\:\times\:$weight$\:\times\:$proportion + Flood engineering measures$\:\times\:$weight$\:\times\:$proportion
Change in inherent resilience of gas system	Rate	Change in inherent resilience of gas system = Hazard level of major hazard sources$\:\times\:$weight$\:\times\:$proportion + Proportion of high-pressure and Sub-HP pipelines$\:\times\:$weight$\:\times\:$proportion+ Pipeline corrosion factors assessment$\:\times\:$weight$\:\times\:$proportion+ Pipeline site protection plan$\:\times\:$weight$\:\times\:$proportion+ Security of critical infrastructure$\:\times\:$weight$\:\times\:$proportion+ Pipeline corrosion protection$\:\times\:$weight$\:\times\:$proportion
Resilience variation of gas system	Rate	Resilience variation of gas system = Resilience of surrounding population distribution$\:\times\:$weight$\:\times\:$proportion$\:+\:$Resilience of Buildings Surrounding Gas Systems$\:\times\:$weight$\:\times\:$proportion+ Resilience of surrounding infrastructure of gas system$\:\times\:$weight$\:\times\:$proportion
Changes in emergency, rescue, and evacuation capabilities	Rate	Changes in emergency, rescue, and evacuation capabilities =Lifeline emergency capability$\:\times\:$weight$\:\times\:$proportion +Emergency rescue capability$\:\times\:$weight$\:\times\:$proportion +Channel evacuation capability$\:\times\:$weight$\:\times\:$proportion
Security of critical infrastructure	Rate	Security of critical infrastructure =Lifeline seismic safety$\:\times\:$weight$\:\times\:$proportion + Emergency infrastructure level$\:\times\:$weight$\:\times\:$proportion
Hazard level of major hazard sources	Rate	Hazard level of major hazard sources = LNG station siting$\:\times\:$weight$\:\times\:$proportion +CNG fueling station siting$\:\times\:$weight$\:\times\:$proportion + Major hazard protection$\:\times\:$proportion$\:\times\:$weight
Regional disaster integrity	Rate	Regional disaster integrity =Flood engineering measures$\:\times\:$weight$\:\times\:$proportion +Geohazard assessment$\:\times\:$weight$\:\times\:$proportion +Geohazard mitigation measures$\:\times\:$weight$\:\times\:$proportion
Pipeline site protection plan	Rate	Pipeline site protection plan=Construction and pipeline signage$\:\times\:$weight$\:\times\:$proportion +Permanent markings$\:\times\:$weight$\:\times\:$proportion +On-site monitoring$\:\times\:$weight$\:\times\:$proportion
Resilience of surrounding population distribution	Rate	Resilience of surrounding population distribution =Proportion of high-pressure and Sub-HP pipelines$\:\times\:$weight$\:\times\:$proportion +Population density$\:\times\:$weight$\:\times\:$proportion+Population-gas system spatial correlation$\:\times\:$weight$\:\times\:$proportion
Resilience of surrounding infrastructure of gas system	Rate	Resilience of surrounding infrastructure of gas system=Proportion of high-pressure and Sub-HP pipelines$\:\times\:$weight$\:\times\:$proportion+Infrastructure-gas system spatial correlation$\:\times\:$weight$\:\times\:$proportion+Infrastructure distribution resilience$\:\times\:$weight$\:\times\:$proportion
Resilience of Buildings Surrounding Gas Systems	Rate	Resilience of Buildings Surrounding Gas Systems=Proportion of high-pressure and Sub-HP pipelines$\:\times\:$weight$\:\times\:$proportion+Building type resilience$\:\times\:$weight$\:\times\:$proportion+Building-gas system spatial correlation$\:\times\:$weight$\:\times\:$proportion
Channel evacuation capability	Rate	Channel evacuation capability=Passage and exit distribution$\:\times\:$weight$\:\times\:$proportion+Passage and exit accessibility$\:\times\:$weight$\:\times\:$proportion +Regional disaster integrity$\:\times\:$weight$\:\times\:$proportion
Lifeline emergency capability	Rate	Lifeline emergency capability =Lifeline response capability$\:\times\:$weight$\:\times\:$proportio+Fire stations distribution$\:\times\:$weight$\:\times\:$proportion +Emergency infrastructure level$\:\times\:$weight$\:\times\:$proportion+Fire water supply$\:\times\:$weight$\:\times\:$proportion
Emergency rescue capability	Rate	Emergency rescue capability =Emergency response teams$\:\times\:$weight$\:\times\:$proportion +Emergency communications and commands$\:\times\:$weight$\:\times\:$proportion+Emergency medical facility$\:\times\:$weight$\:\times\:$proportion

**Fig. 3:**
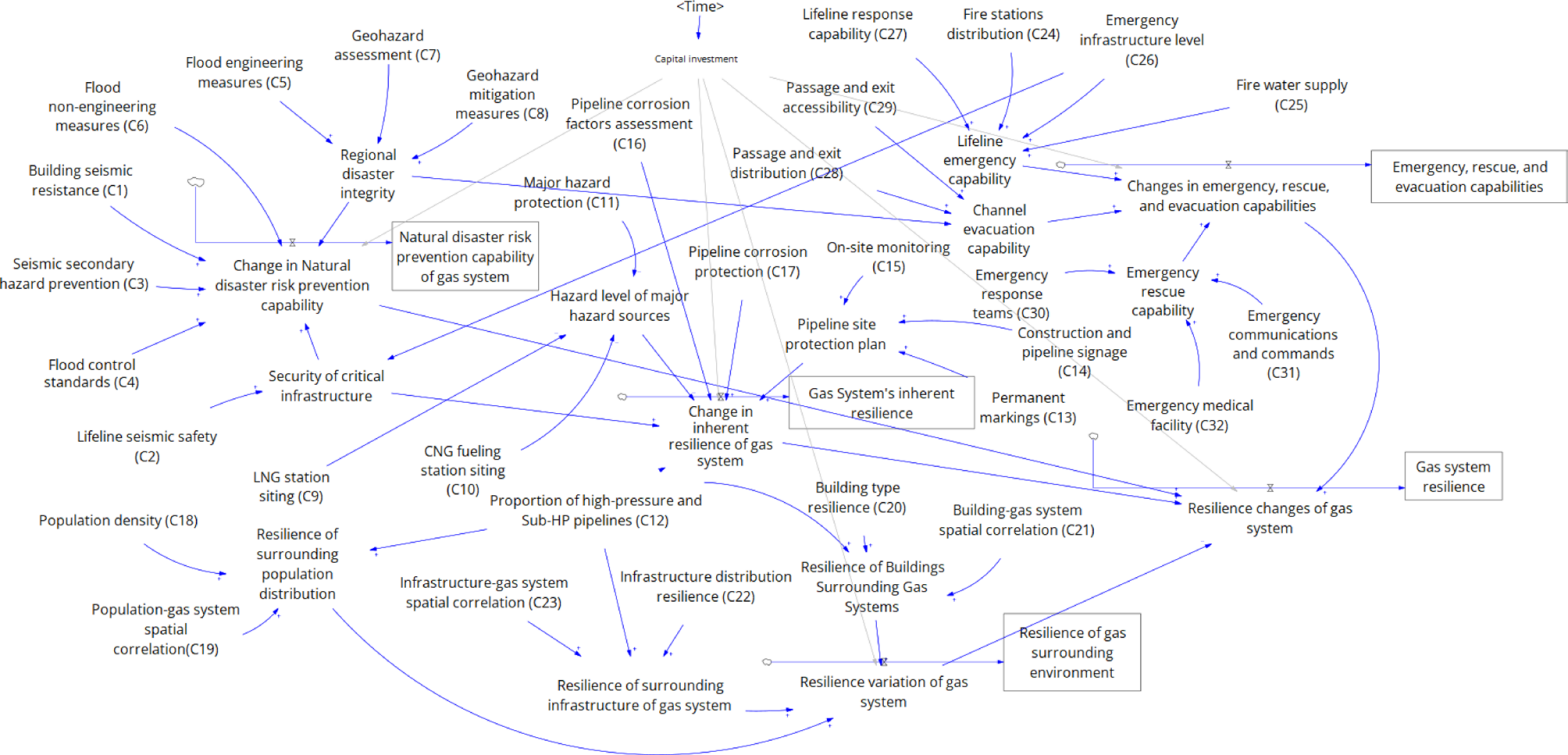
The stock and flow diagram of risk factor assessment for gas infrastructures.

As an example, the fully specified equation for Lifeline emergency capability is: *Lifeline emergency capability* = (Lifeline response capability × 0.497 × 0.0885) + (Fire stations distribution × 0.248 × 0.0885) + (Emergency infrastructure level × 0.166 × 0.0885) + (Fire water supply × 0.089 × 0.0885). Where the first factor in each product (e.g., 0.497) is the proportion weight from Tab.le 7, and the second factor (0.0885) is the overall variable weight. Similarly, the Vensim software was utilized to construct this SD flow diagram. Therefore, the causal loop diagram was translated into a flow diagram in Fig. 3, incorporating state variables (levels), rate variables (flows), and auxiliary variables. This figure representation more effectively captures the system’s dynamic behavior. In addition, uncertainty is handled in two ways. First, parameter uncertainty is captured through the use of multiple expert judgments, and the range of their scores provides implicit uncertainty bounds. Second, sensitivity analysis explicitly tests how outcomes vary with parameter changes, identifying which uncertainties matter most. However, we do not implement probabilistic simulation to propagate uncertainty distributions; this is acknowledged as a limitation and future direction in Sect.  4.

## Case study

### Background

**Fig. 4:**
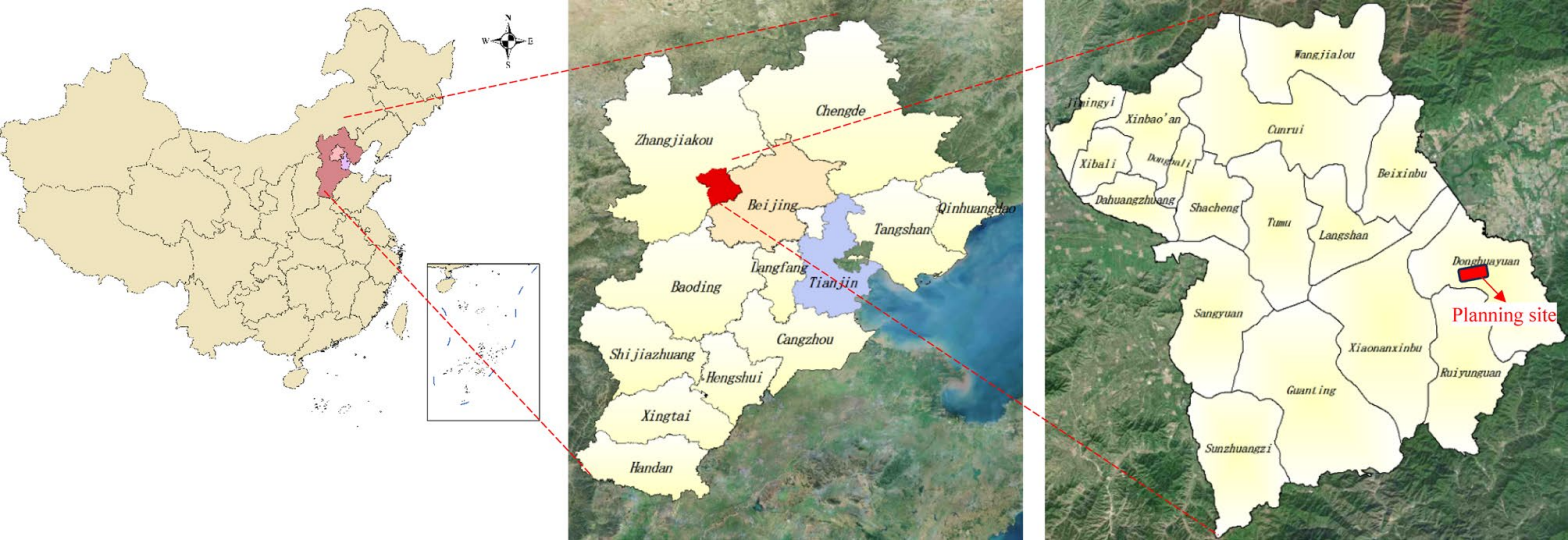
National, provincial and county-level locations of the study area. (sourced from related planning and drawn by the authors.)

**Fig. 5:**
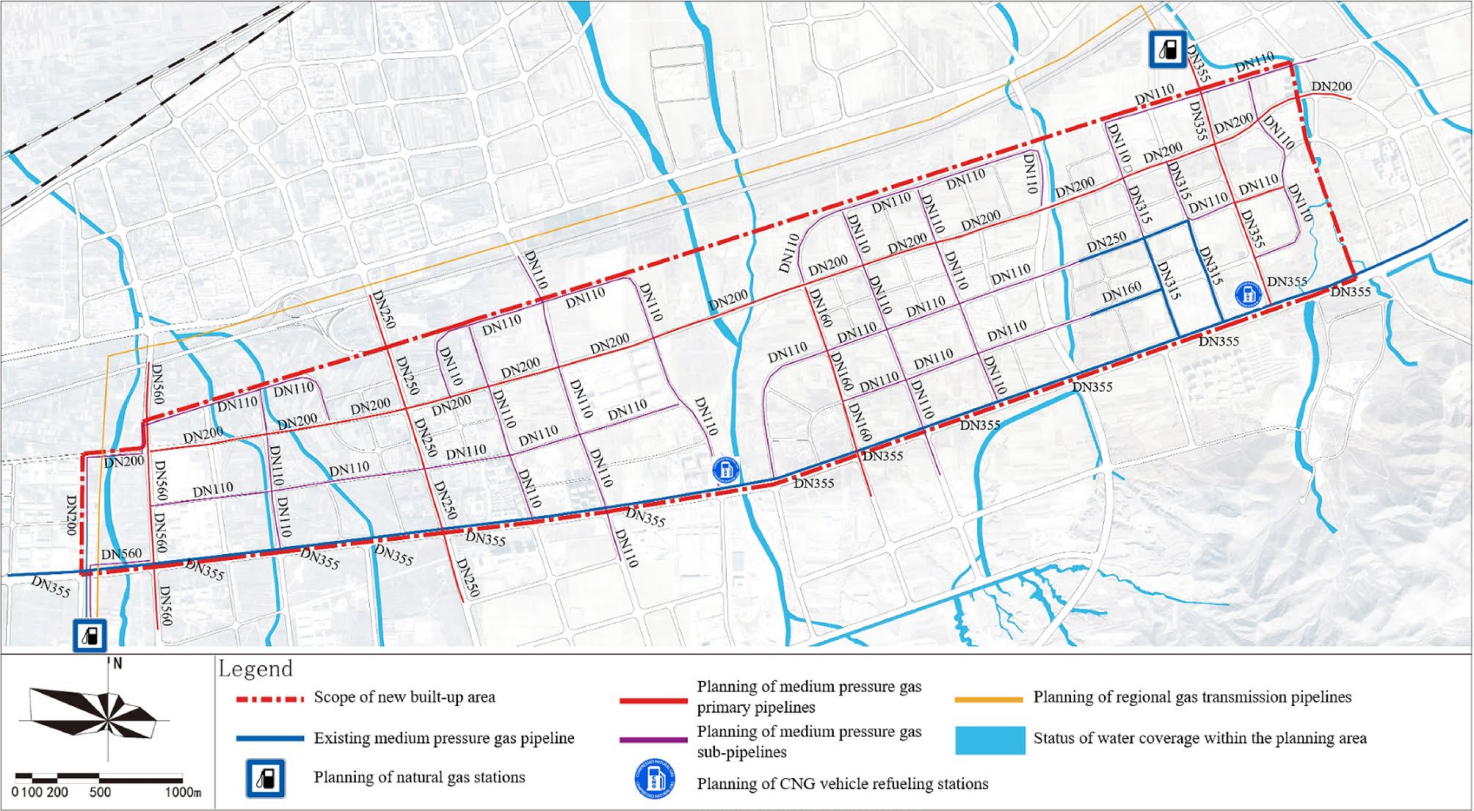
Gas planning of the study area sourced from Huailai CG [53] and related planning, drawn by the authors.

A case study is conducted on a real municipal gas pipeline network in China’s Huailai Garden Ecological Science and Technology New Zone, located in Huailai County, Zhangjiakou City, near Beijing. As shown in Figs. 4 and 5, an 8 km × 1.2 km urban area with four categories of gas pipelines is selected for analysis. Primary pipelines (red lines) traverse primarily industrial zones, while sub-pipelines (purple) and low-pressure (yellow) pipelines serve residential and commercial areas. Water coverage areas connect to planning spaces designated as emergency shelters. The planning of this area has been publicly disclosed^53^.

This case study is representative due to the following characteristics:

(1) It is a newly developed urban area with a comprehensive plan, functioning as a big data industry base that balances work and residence. The area includes diverse land uses such as municipal facilities, residential, commercial, industrial, educational, hospital, and urban green spaces.

(2) The area has a complete gas planning system, featuring conventional gas pipelines of varying diameters, natural gas stations, and vehicle refueling stations, all of which are potential risk sources related to the gas system.

(3) The urban area includes critical public service facilities such as schools and hospitals, requiring special protection, along with emergency evacuation routes and urban green spaces serving as emergency shelters. These functionalities exhibit interdependence.

(4) The project is divided into two stages: original planning and adjusted planning. The gas planning aligns with the adjusted planning phase. During evaluation, seven experts compared the original and adjusted planning, focusing on the adjusted phase and scoring based on the established index system. Land adjustments in the planning altered the layout and scale of residential, public, and commercial facilities, leading to corresponding changes in gas system risks. Therefore, the land-use changes in the adjusted planning, such as the relocation of residential areas away from high-pressure pipelines, directly inform the expert scoring for spatial correlation indicators like C19. This linkage ensures that the abstract model variables are grounded in concrete planning changes.

### Calculation of indicator weights and consistency analysis

**Fig. 6:**
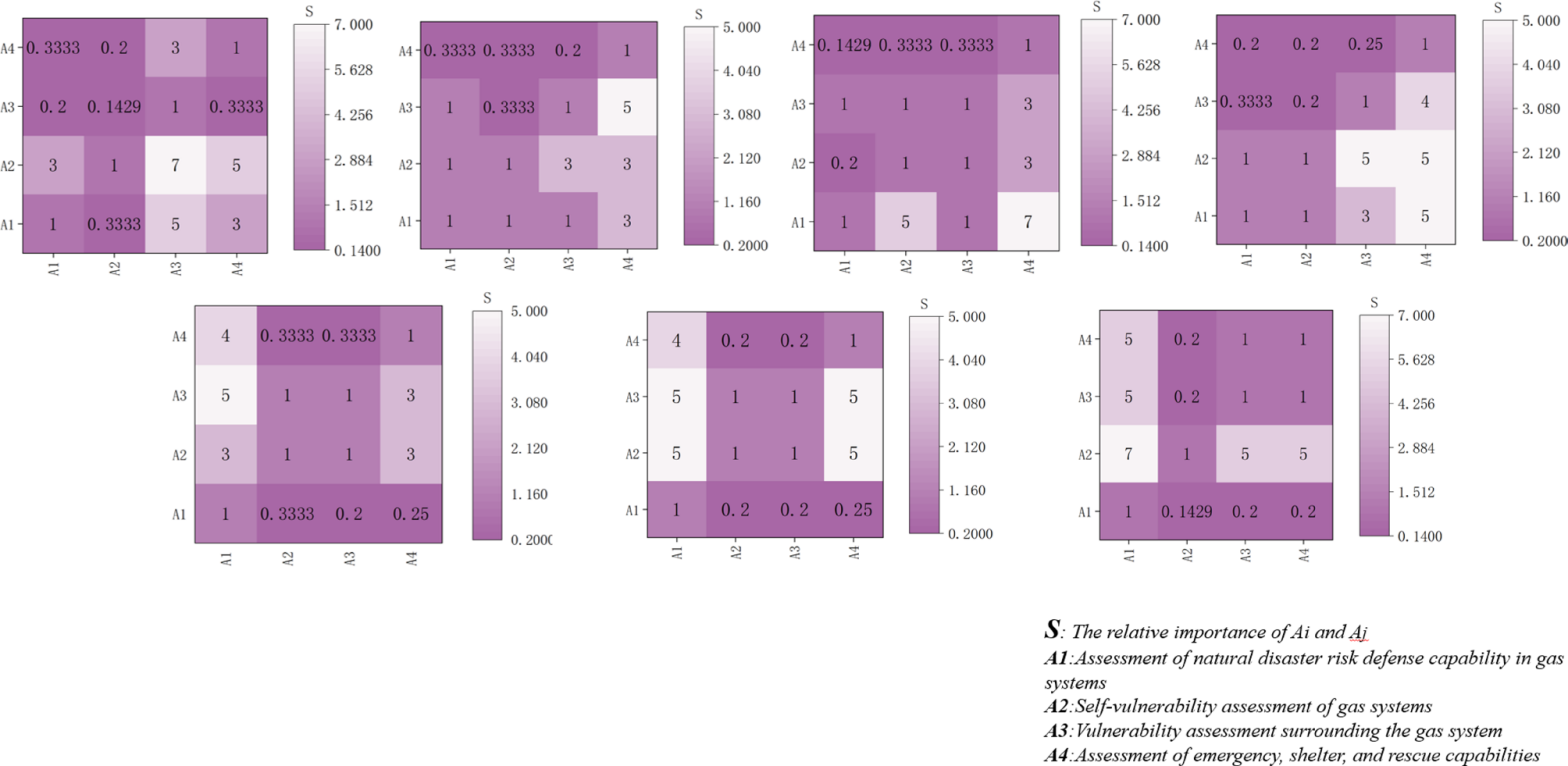
Heat map of indicator matrix from experts’ judgment.

Calculation of indicator weights and consistency analyses need to be carried out prior to the risk evaluation. According to the indicator system of regional gas system risk evaluation, Saaty’s 1–9 scale method was used to design the questionnaire, and the experts were invited to compare the indicators at all levels two by two to arrive at the judgment matrix. The judgment matrix of seven experts on the first level indicators is shown in Fig. 6.

According to the seven judgment matrices shown in the figure, the consistency ratios of the judgment matrices were calculated to be 0.0444, 0.0991,0.0947, 0.0732, 0.0727, 0.0985 and 0.0794, all of which passed the consistency test. After obtaining the weights of various indicators, take the average to obtain the final weights, as shown in Table 6.

**Table 6:** Weights of risk evaluation indicators for gas systems.

Level-1 indicators and corresponding weights		Level-2 indicators and corresponding weights		Weighting of Level-3 versus Level-2	Level-3 indicators and corresponding weights		Weighting of Level-3 versus Level-2
A1	0.1399	B1	0.0342	0.3129	C1	0.0216	0.632
					C2	0.0028	0.082
					C3	0.0098	0.286
		B2	0.0410	0.3751	C4	0.0170	0.499
					C5	0.0106	0.311
					C6	0.0065	0.190
		B3	0.0341	0.3120	C7	0.0149	0.363
					C8	0.0261	0.637
A2	0.4494	B4	0.1546	0.3378	C9	0.0446	0.288
					C10	0.0309	0.199
					C11	0.0791	0.513
		B5	0.1269	0.2773	C12	0.0789	0.622
					C13	0.0480	0.378
		B6	0.0996	0.2176	C14	0.0711	0.714
					C15	0.0285	0.286
		B7	0.0766	0.1673	C16	0.0408	0.533
					C17	0.0358	0.467
A3	0.2634	B8	0.1682	0.6302	C18	0.0433	0.257
					C19	0.1249	0.743
		B9	0.0524	0.1963	C20	0.0262	0.500
					C21	0.0262	0.500
		B10	0.0463	0.1735	C22	0.0198	0.378
					C23	0.0265	0.572
A4	0.1471	B11	0.0298	0.1991	C24	0.0219	0.735
					C25	0.0079	0.265
		B12	0.0587	0.3921	C26	0.0147	0.251
					C27	0.0440	0.749
		B13	0.0425	0.2839	C28	0.0308	0.725
					C29	0.0117	0.275
		B14	0.0187	0.1249	C30	0.0078	0.412
					C31	0.0067	0.372
					C32	0.0042	0.226

### Parameter setting

According to calculated indicator weights, the relative importance of each indicator within both the primary system and its respective subsystem was determined. Within the integrated system dynamics framework, composite variables were computed using weighted summations, e.g., *Evacuation Corridor Capacity = (Passage Accessibility × Weight × Proportion) + (Passage Distribution Density × Weight × Proportion)*. Proportions for component indicators, e.g., passage accessibility and passage distribution density were derived through normalization of existing weight data.

7 $$\:\begin{array}{c}W=\frac{xi}{\sum\:_{n}^{1}xn}\:\end{array}$$

where$\:\:xi$ is weight of the indicator, $\:\sum\:_{n}^{1}xn$ is the sum of the internal indicator weights of the variable. The variable, weight and proportion are shown in Table 7.

**Table 7:** The variable, weight and proportion setting.

Variable and weight	Sub variable	Proportion
Change in natural disaster risk prevention capability(0.125)	Building seismic resistance	0.173
	Regional disaster integrity	0.421
	Security of critical infrastructure	0.14
	Secondary disaster defense	0.078
	Flood control standards	0.136
	Flood non-engineering measures	0.052
Change in system’s inherent resilience(0.4754)	Hazard level of major hazard sources	0.325
	Proportion of high-pressure and Sub-HP pipelines	0.166
	Pipeline corrosion protection	0.075
	Pipeline site protection plan	0.311
	Security of critical infrastructure	0.037
	Pipeline corrosion factors assessment	0.086
Resilience variation of gas system(0.5038)	Resilience of surrounding population distribution	0.49
	Resilience of surrounding infrastructure of gas system	0.249
	Resilience of Buildings Surrounding Gas Systems	0.261
Changes in emergency, rescue, and evacuation capabilities(0.2023)	Lifeline emergency capability	0.437
	Channel evacuation capability	0.470
	Emergency rescue capability	0.093
Security of critical infrastructure(0.0175)	Lifeline project seismicity	0.16
	Emergency infrastructure level	0.84
Hazard level of major hazard sources(0.1546)	LNG station siting	0.288
	CNG fueling station siting	0.2
	Major hazard protection	0.512
Regional disaster integrity(0.0526)	Flood engineering measures	0.202
	Geohazard assessment	0.497
	Geohazard mitigation measures	0.301
Pipeline site protection plan(0.1476)	On-site monitoring	0.193
	Construction and pipeline signage	0.482
	Permanent markings	0.325
Resilience of surrounding population distribution (0.2471)	Proportion of high-pressure and Sub-HP pipelines	0.319
	Population density	0.175
	Population-gas system spatial correlation	0.506
Resilience of surrounding infrastructure of gas system (0.1254)	Proportion of high-pressure and Sub-HP pipelines	0.629
	Infrastructure-gas system spatial correlation	0.211
	Infrastructure distribution resilience	0.16
Resilience of Buildings Surrounding Gas Systems (0.1313)	Proportion of high-pressure and Sub-HP pipelines	0.6
	Building type resilience	0.2
	Building-gas system spatial correlation	0.2
Channel evacuation capability (0.0951)	Passage and exit accessibility	0.123
	Passage and exit distribution	0.324
	Regional disaster integrity	0.553
Lifeline emergency capability(0.0885)	Fire stations distribution	0.248
	Lifeline response capability	0.497
	Emergency infrastructure level	0.166
	Fire water supply	0.089
Emergency rescue capability (0.0187)	Emergency response teams	0.417
	Emergency medical facility	0.358
	Emergency communications and commands	0.225

Given the predominantly qualitative nature of variables, expert scoring was employed for data acquisition. All variables were assigned values on a normalized 0–1 scale, where 0 represents the initial (least developed) state and 1 signifies the optimal (most developed) state of evolution. Expert scores for each indicator were averaged using the formula:

8 $$\:\begin{array}{c}\:Xi=\frac{X1+X2+X3+\dots\:+Xn}{n}\end{array}$$

where *Xi* is the average value of the data, *X1*,* X2…… Xn* are the ratings of different experts on the indicators, and n is the number of experts.

Subsequently, normalize the data:

9 $$\:\begin{array}{c}y=\frac{Xi-Xmin}{Xmax-Xmin}\end{array}$$

where *Xi* is the average value of the data, *Xmin* is 1, *Xmax* is 5. The normalized data is shown in Table 8.

**Table 8:** The indicators and initial value setting.

Indicators	Initial value	Indicators	Initial value
Building seismic resistance	0.785	Construction and pipeline signage	0.75
Seismic secondary hazard prevention	0.6075	Population density	0.6425
Flood control standards	0.6425	Population-gas system spatial correlation	0.535
Flood non-engineering measures	0.5725	Infrastructure-gas system spatial correlation	0.6775
Proportion of high-pressure and Sub-HP pipelines	0.6071	Infrastructure distribution resilience	0.6775
Pipeline corrosion protection	0.7857	Building type resilience	0.715
Major hazard protection	0.6425	Building-gas system spatial correlation	0.6425
Lifeline seismic safety	0.8225	Passage and exit accessibility	0.785
Pipeline corrosion factors assessment	0.715	Passage and exit distribution	0.6425
LNG station siting	0.465	Fire stations distribution	0.6775
CNG fueling station siting	0.5	Lifeline response capability	0.785
Permanent markings	0.7857	Emergency infrastructure level	0.785
Flood engineering measures	0.6775	Fire water supply	0.8225
Geohazard mitigation measures	0.75	Emergency response teams	0.715
Geohazard assessment	0.75	Emergency medical facility	0.715
On-site monitoring	0.7143	Emergency communications and commands	0.715

### Sensitivity analysis

#### Analysis methodology

This article conducts simulations over a period of 100 months, using the “Model” and “Settings” in the Vensim program to define the initial and termination times, as well as the time step. Firstly, it should be clarified that the 100-month simulation period is not strictly a “long-term prediction” of the resilience of urban gas infrastructure, but rather a dynamic analysis window designed based on research objectives and system characteristics. The core purpose is to capture the differential impacts and sensitivities of risk factors. Furthermore, the selection of 20% as the initial disturbance amplitude for the indicators is based on semi-structured interviews with 7 industry experts regarding initial indicator values. This approach aligns with the methodological consensus in infrastructure risk sensitivity analysis, balancing sensitivity identification with realistic intervention space. This magnitude generates quantifiable elastic responses in the system dynamics -based framework, allowing for the accurate identification of key indicators. It should be noted that the 20% increase is a standardized perturbation in sensitivity analysis, not a claim that such improvements are directly achievable. For practical interpretation, a 20% improvement in an abstract indicator like building seismic resistance (C1) could correspond to a specific planning action, such as upgrading from standard code compliance to an enhanced seismic design category. This mapping from abstract percentage to concrete action helps translate sensitivity rankings into actionable planning priorities.

To test the robustness of the findings to the chosen time horizon, the sensitivity rankings derived from the 100-month simulation were compared with those from 50-month and 200-month horizons. The ranking of the top five most sensitive indicators remained identical across all three horizons, with only minor variations (± 0.3%) in the magnitude of resilience changes. This consistency confirms the stability of the key findings with respect to simulation length.

Similarly, to assess the influence of the selected disturbance magnitude, the sensitivity analysis was repeated using 10% and 30% disturbance levels for a subset of the top indicators. The relative ranking of these indicators was preserved, with Spearman’s rank correlation coefficients exceeding 0.95 between the 20% ranking and the alternative rankings. This high correlation indicates that the identification of high-sensitivity factors is robust to the chosen disturbance magnitude.

**Fig. 7:**
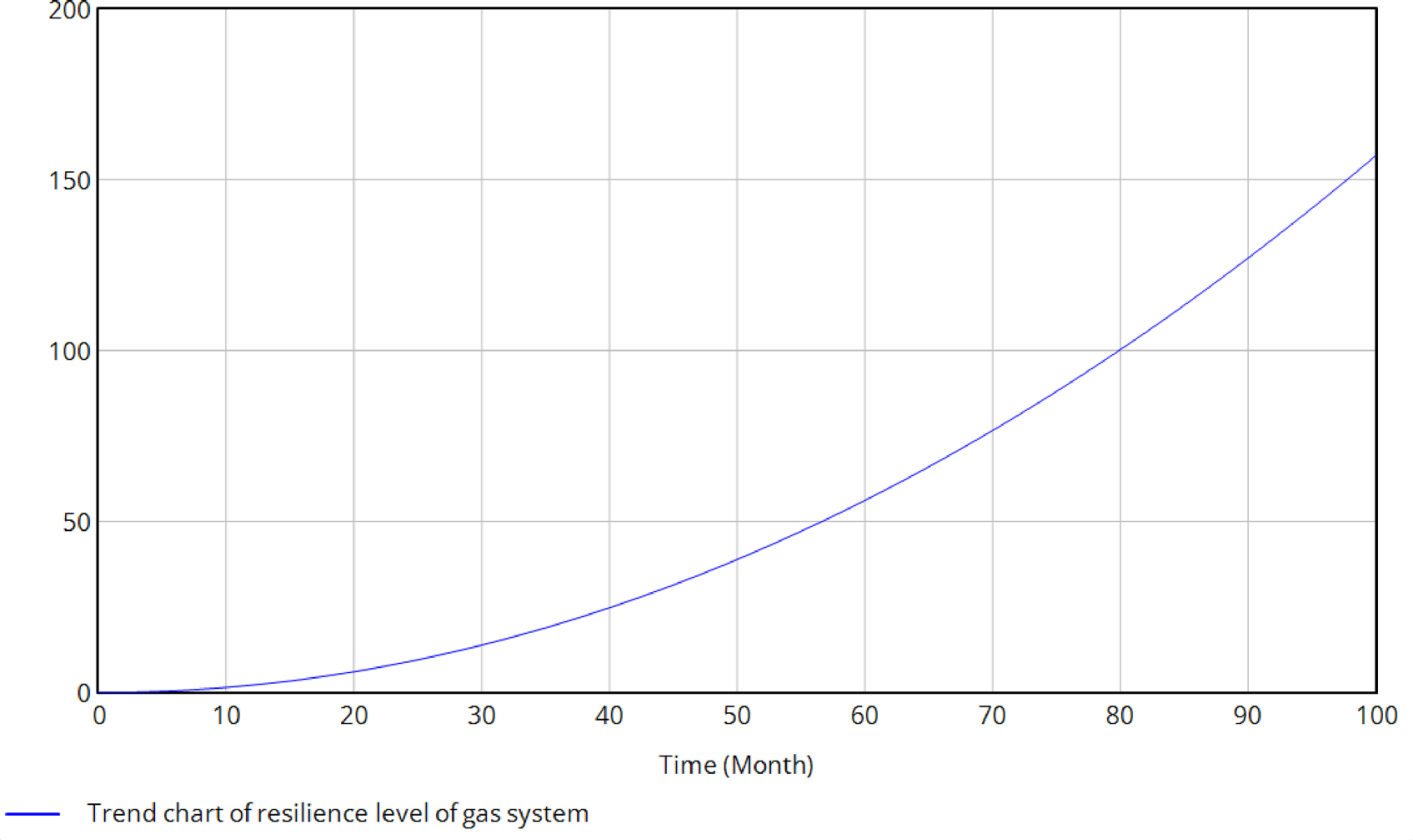
Resilience level trend of gas system.

**Fig. 8:**
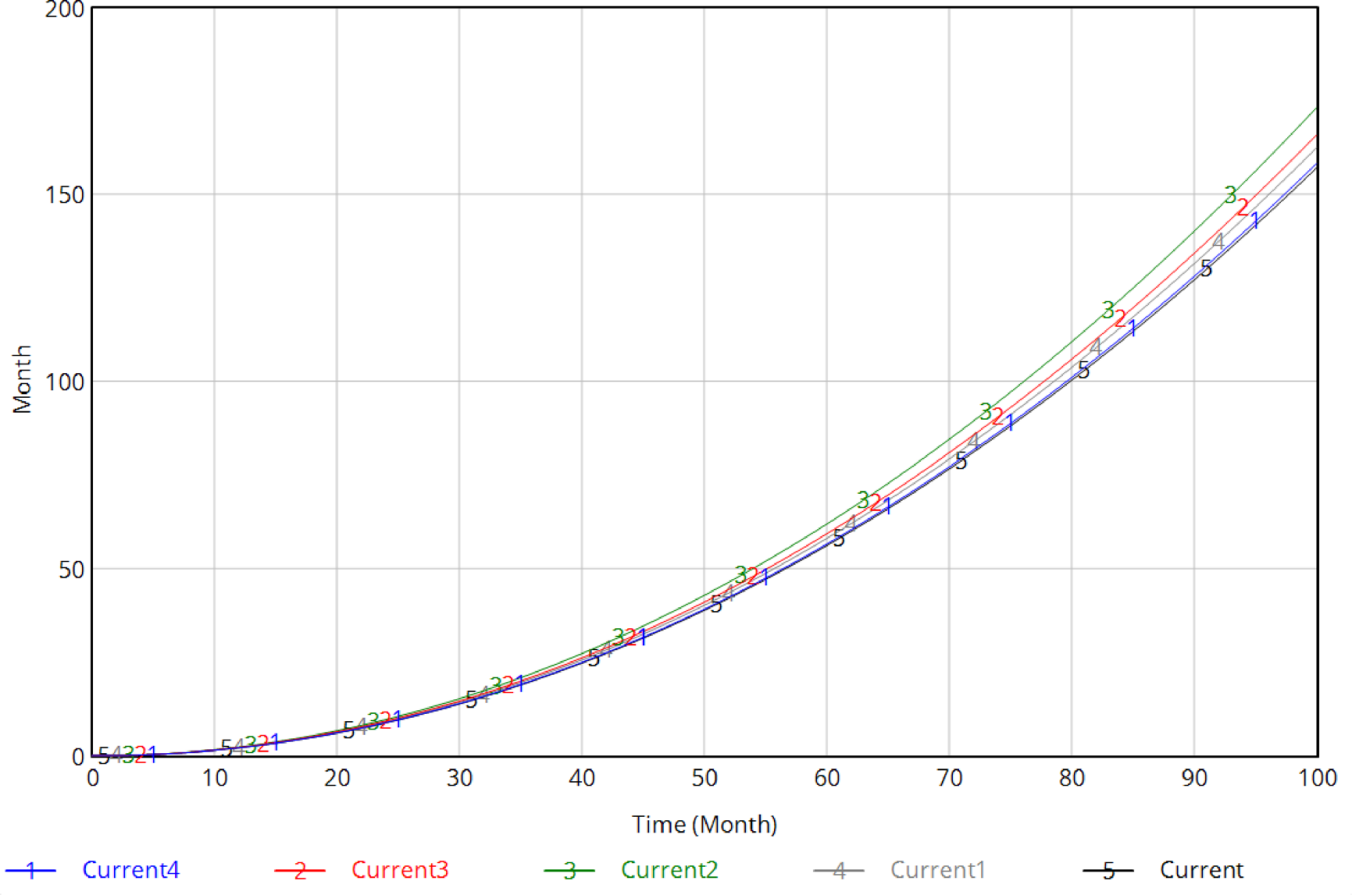
Sensitivity trend of gas system resilience.

Gas system resilience dynamics were simulated using the developed system dynamics-based framework, generating resilience evolution trend figure, shown in Fig. 7. To quantify the influence of key subsystems: Natural disaster resilience, system’s inherent resilience, resilience of gas surrounding environment, and emergency, rescue, and evacuation capabilities, the initial value of each subsystem was independently increased by 20% while holding other factors constant. The resulting change in system resilience was analyzed. Sensitivity increased with the magnitude of impact on resilience, with the most sensitive factors identified as critical leverage points. Results are presented in Table 9; Fig. 8.

**Table 9:** Varying patterns of sensitive impact factors.

Patterns	Description of changes	Maximum value
current	keeping all factors unchanged.	157.131
current 1	Increase the natural disaster risk prevention capability of gas system by 20%, while keeping other factors unchanged.	162.5
current 2	Increase the gas system’s inherent resilience by 20%, while keeping other factors unchanged.	173.23
current 3	Increase the resilience of gas surrounding environment by 20%, while keeping other factors unchanged.	165.93
current 4	Increase the emergency, rescue, and evacuation capabilities by 20%, while keeping other factors unchanged.	158.289

Simulation results indicate a gradual increase in gas system resilience over time. Furthermore, sensitivity analysis revealed the highest sensitivity to system’s inherent resilience, followed by resilience of gas surrounding environment and natural disaster resilience. Emergency, rescue, and evacuation capabilities exhibited the lowest sensitivity. This hierarchy arises because safety considerations, e.g., pipeline routing, hazard source protection, land-use compatibility. There are foundational factors of system’s inherent and surrounding resilience addressed during initial planning phases, whereas emergency response capabilities typically develop incrementally through subsequent infrastructure investments. Further sensitivity analysis was conducted on individual factors within each subsystem.

The main methods of analysis are conducting factor-level sensitivity testing first. For example, the initial value of a single indicator increased by 20%, while all other factors remained unchanged. Subsequently, the modified model output was compared with current. The magnitude of deviation quantified the factor’s sensitivity. The results for each modification are run and viewed, such as current 1, current 2. Meanwhile, the change rate was calculated as: Change Rate (%) = [(Modified Mean – Current Mean) / Current Mean] × 100.

#### Subsystem of natural disaster risk prevention

**Fig. 9:**
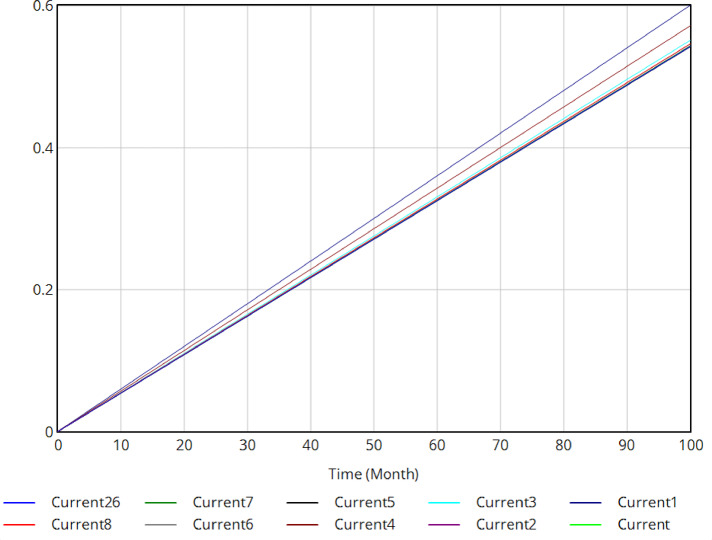
Changes in natural disaster resilience after sequentially changing of subsystem factors.

**Fig. 10:**
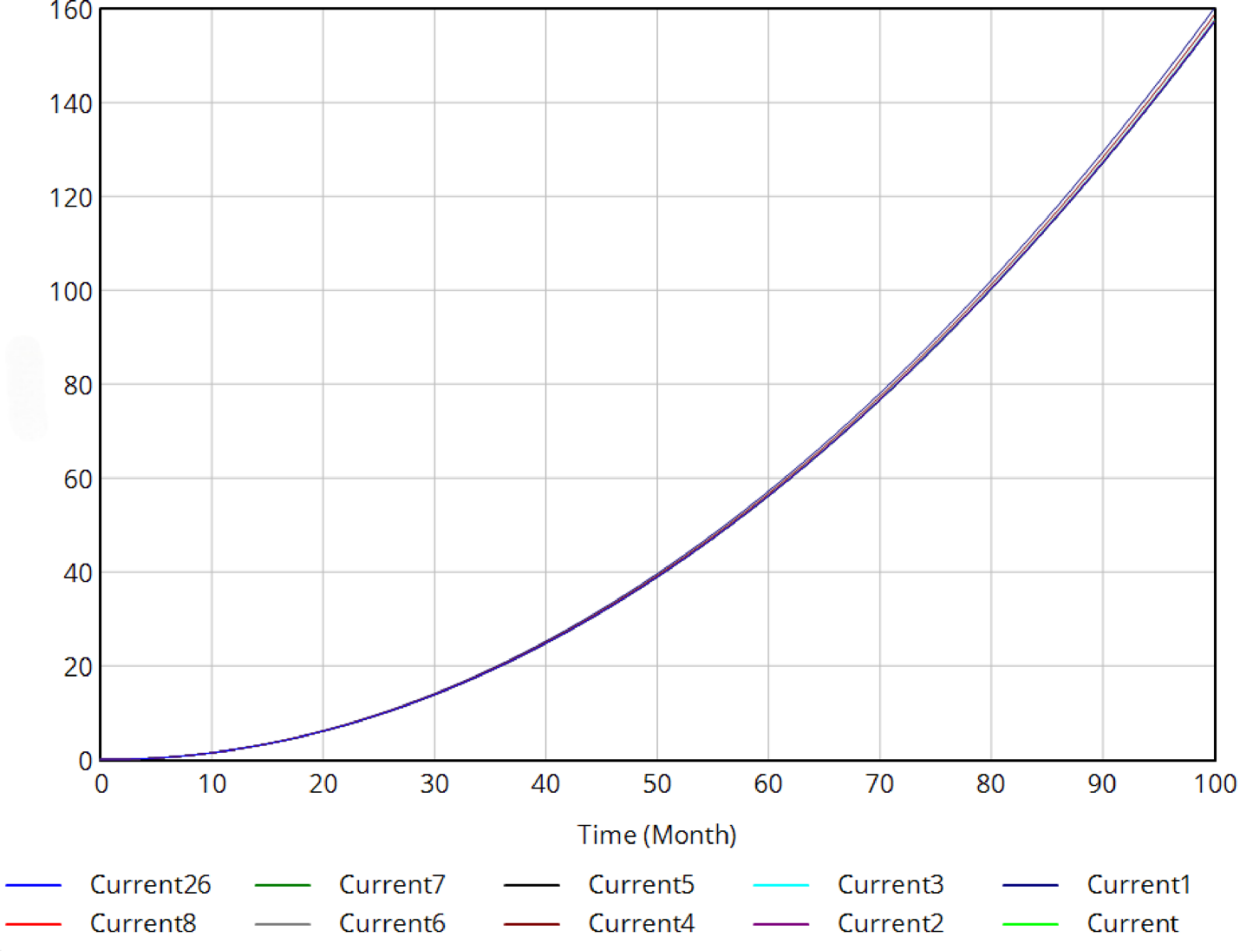
Changes in integrated resilience after sequentially changing factors of the natural disaster resistance subsystem.

In the simulation process, the current represents natural disaster resilience under original parameters. Similarly, the modified runs of current1-current26 represent natural disaster resilience after individually increasing Building Seismic Resistance (C1), Lifeline Seismic Safety (C2), Seismic Secondary Hazard Prevention (C3), Flood Control Standard (C4), Flood Engineering Measures (C5), Flood Non-Engineering Measures (C6), Geohazard Assessment (C7), Geohazard Mitigation Measures (C8), and Emergency Infrastructure Level (C26) by 20%. Meanwhile, the initial value of each other index is unchanged. The changes in natural disaster resilience of the gas system are shown in Fig. 9, and the resilience changes of the gas system are shown in Fig. 10. Furthermore, the sensitivity analysis of natural disaster risk prevention capability is shown in Table 10.

**Table 10:** Sensitivity analysis of natural disaster risk prevention capability.

Status Number	Measures	Natural disaster risk prevention capability	Growth rate	Gas system resilience	Growth rate
Current	Keep current status	0.271	-	52.377	-
C1	Increase 20%	0.301	11.07%	53.345	1.85%
C2	Increase 20%	0.271	≈ 0%	52.377	≈ 0%
C3	Increase 20%	0.276	1.85%	52.530	0.29%
C4	Increase 20%	0.286	5.53%	52.867	0.94%
C5	Increase 20%	0.272	0.37%	52.388	≈ 0%
C6	Increase 20%	0.273	0.74%	52.441	0.12%
C7	Increase 20%	0.272	0.37%	52.403	0.05%
C8	Increase 20%	0.273	0.74%	52.452	0.14%
C26	Increase 20%	0.272	0.37%	52.387	≈ 0%

#### Subsystem of gas system’s inherent resilience

**Fig. 11:**
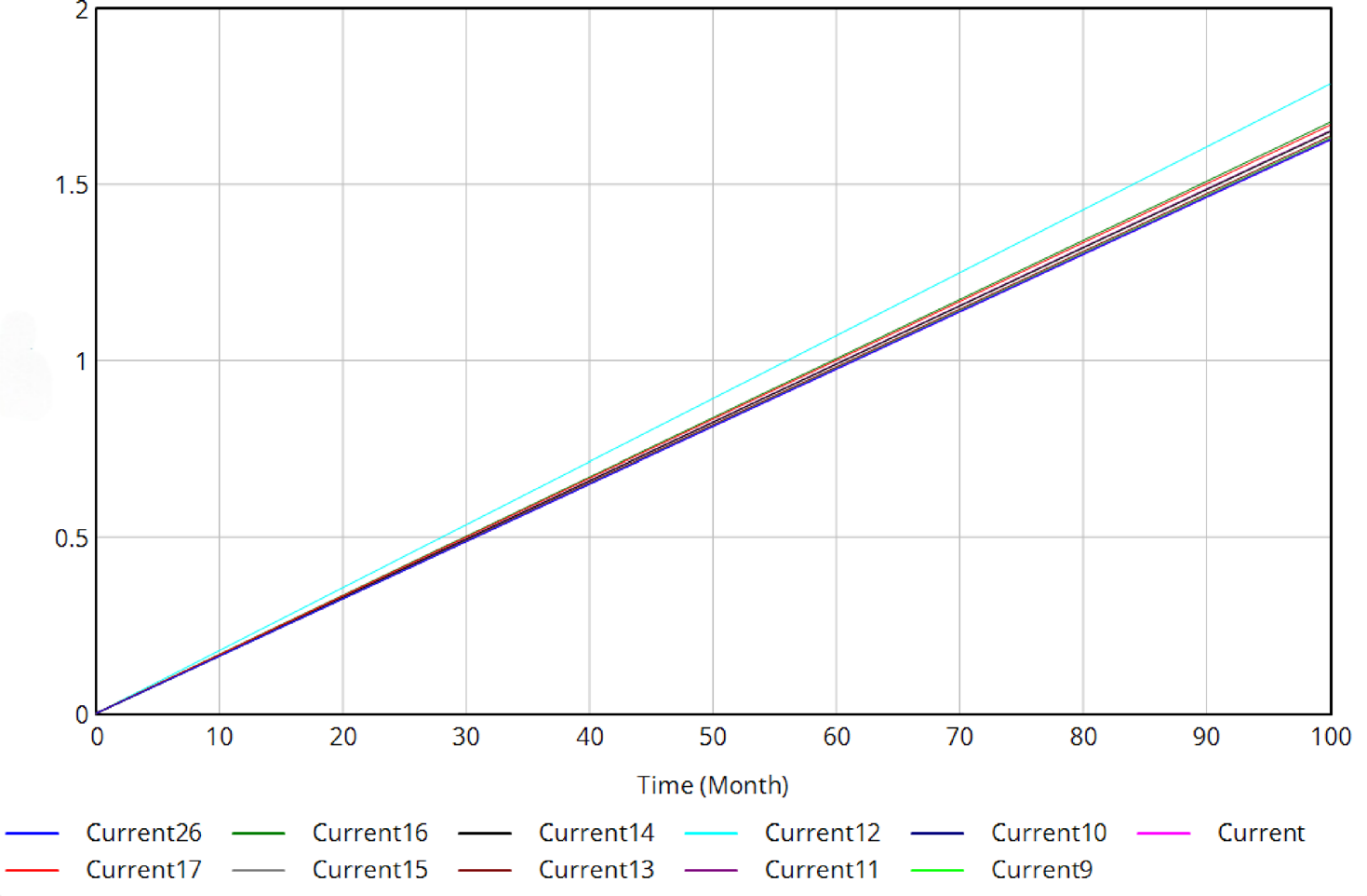
Changes in gas system’s inherent resilience after sequentially changing of subsystem factors.

**Fig. 12:**
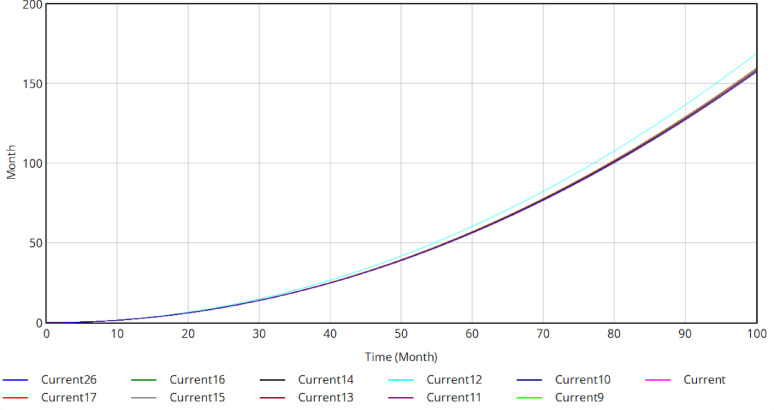
Changes in integrated resilience after sequentially changing factors of the system’s inherent resilience subsystem.

In this simulation process, the current represents gas system’s inherent resilience under original parameters. Similarly, the modified runs of current9-current26 represent system’s inherent resilience after individually increasing LNG Station Siting (C9), CNG Station Siting (C10), Major Hazard Protection (C11), Proportion of high-pressure and Sub-HP Pipeline (C12), Permanent Markings (C13), Construction and Pipeline Signage (C14), On-site Monitoring (C15), Pipeline corrosion factors assessment (C16), Pipeline Corrosion Protection (C17), and Emergency Infrastructure Level (C26) by 20%. Meanwhile, the initial value of each other index is unchanged. The changes in gas system’s inherent resilience are shown in Fig. 11, and the resilience changes of the gas system are shown in Fig. 12. Furthermore, the sensitivity analysis of natural disaster risk prevention capability is shown in Table 11.

**Table 11:** Sensitivity analysis of gas system’s inherent resilience.

Status Number	Measures	Gas system’s inherent resilience	Growth rate	Gas system resilience	Growth rate
Current	Keep current status	0.813	-	52.377	-
C9	Increase 20%	0.816	0.37%	52.476	0.19%
C10	Increase 20%	0.815	0.25%	52.428	0.10%
C11	Increase 20%	0.826	1.60%	52.808	0.82%
C12	Increase 20%	0.893	9.84%	56.247	7.39%
C13	Increase 20%	0.819	0.74%	52.563	0.36%
C14	Increase 20%	0.825	1.48%	52.766	0.74%
C15	Increase 20%	0.815	0.25%	52.437	0.11%
C16	Increase 20%	0.838	3.08%	53.205	1.58%
C17	Increase 20%	0.834	2.58%	53.073	1.33%
C26	Increase 20%	0.813	≈ 0%	52.387	≈ 0%

#### Subsystem of resilience in gas surrounding environment

**Fig. 13:**
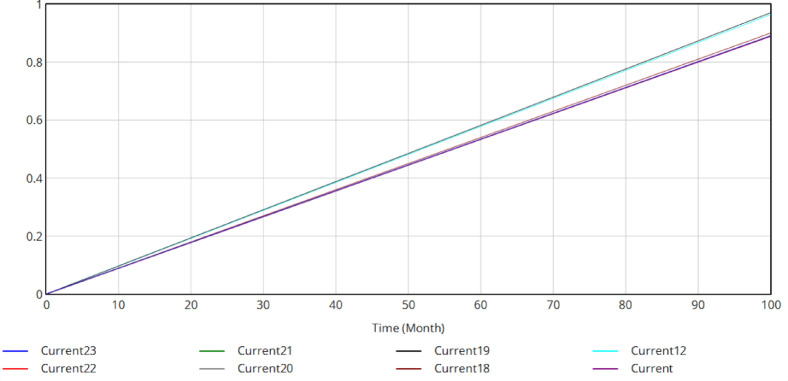
Changes in resilience of gas surrounding environment after sequentially changing of subsystem factors.

**Fig. 14:**
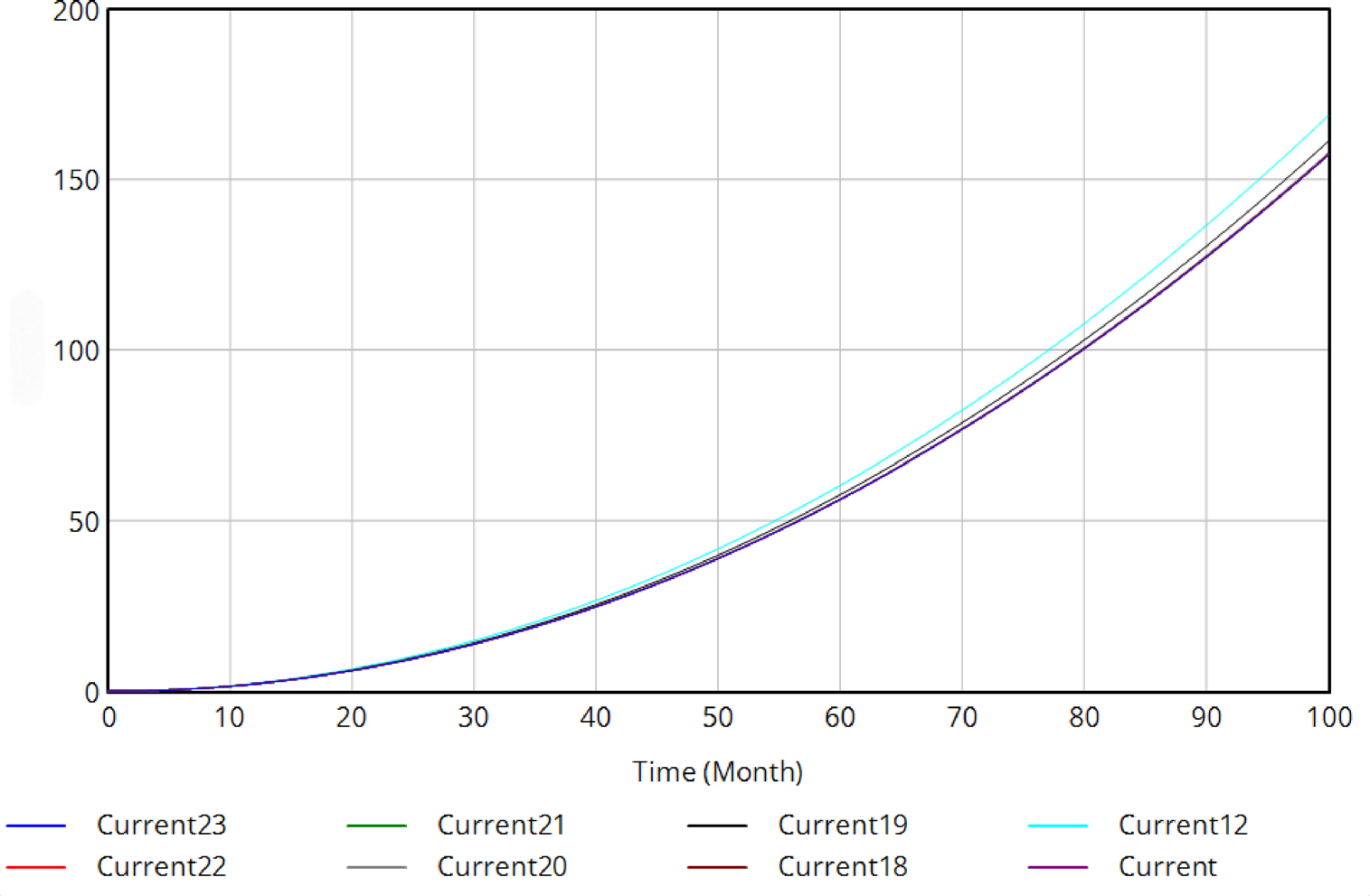
Changes in integrated resilience after sequentially changing factors of the gas surrounding environment subsystem.

In this simulation process, the current represents resilience in gas surrounding environment under original parameters. Similarly, the modified runs of current12-current23 represent resilience in gas surrounding environment after individually increasing Proportion of high-pressure and Sub-HP pipeline (C12), Population density (C18), Population-gas system spatial correlation (C19), Building type resilience (C20), Building-gas system spatial correlation (C21), Infrastructure distribution resilience (C22), and Infrastructure-gas system spatial correlation (C23) by 20%. Meanwhile, the initial value of each other index is unchanged. The changes in resilience in gas surrounding environment are shown in Fig. 13, and the resilience changes of the gas system are shown in Fig. 14. Furthermore, the sensitivity analysis of resilience in gas surrounding environment is shown in Table 12.

**Table 12:** Sensitivity analysis of resilience in gas surrounding environment.

Status Number	Measures	Resilience in gas surrounding environment	Growth rate	Gas system resilience	Growth rate
Current	Keep current status	0.444	-	52.377	-
C12	Increase 20%	0.482	8.56%	56.247	7.39%
C18	Increase 20%	0.450	1.35%	52.572	0.37%
C19	Increase 20%	0.485	9.23%	53.728	2.58%
C20	Increase 20%	0.446	0.45%	52.419	0.08%
C21	Increase 20%	0.446	0.45%	52.415	0.07%
C22	Increase 20%	0.445	0.23%	52.399	0.04%
C23	Increase 20%	0.446	0.45%	52.416	0.07%

#### Subsystem of emergency, rescue, and evacuation capabilities

**Fig. 15:**
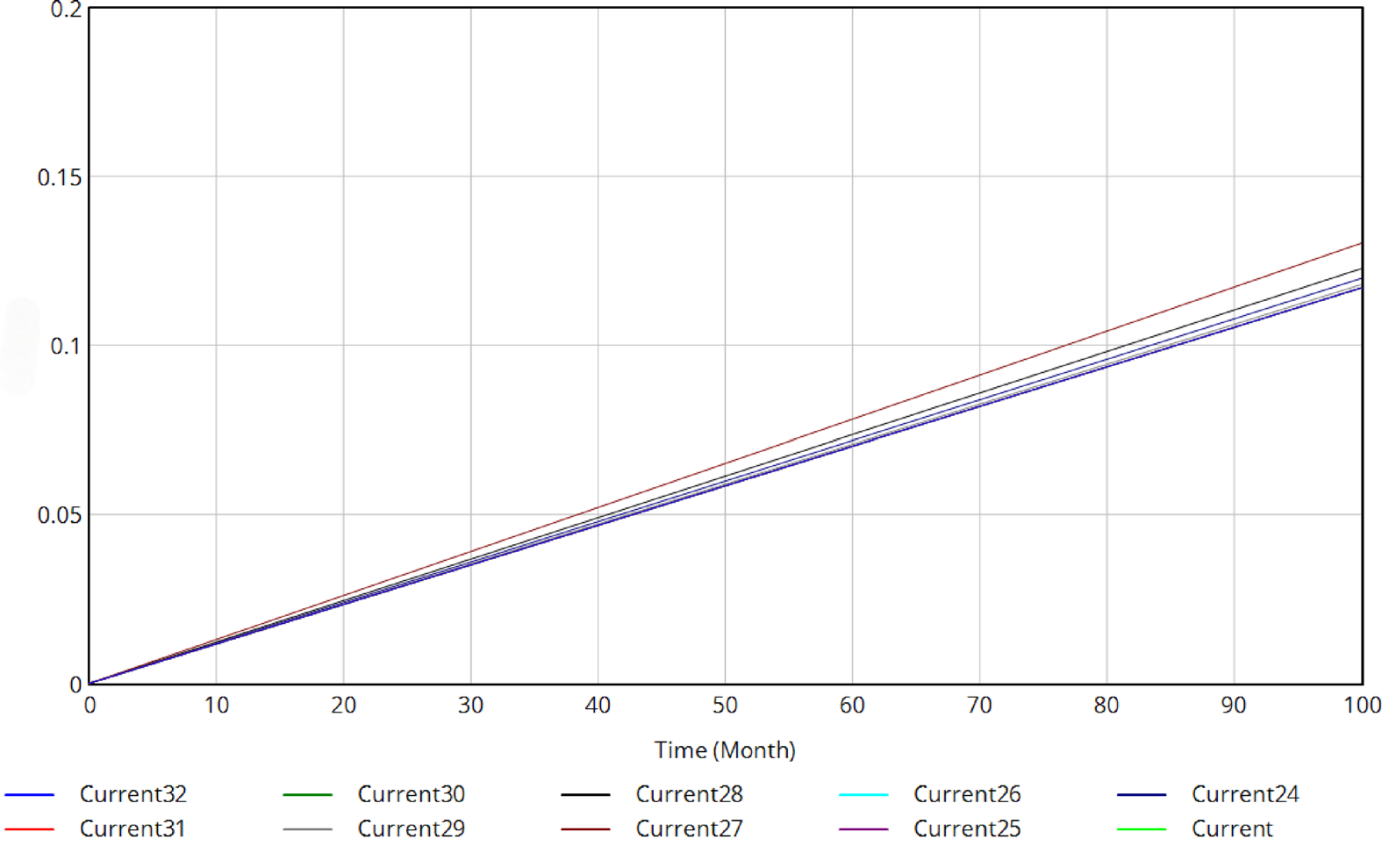
Changes in emergency, rescue, and evacuation capabilities after sequentially changing of subsystem factors.

**Fig. 16:**
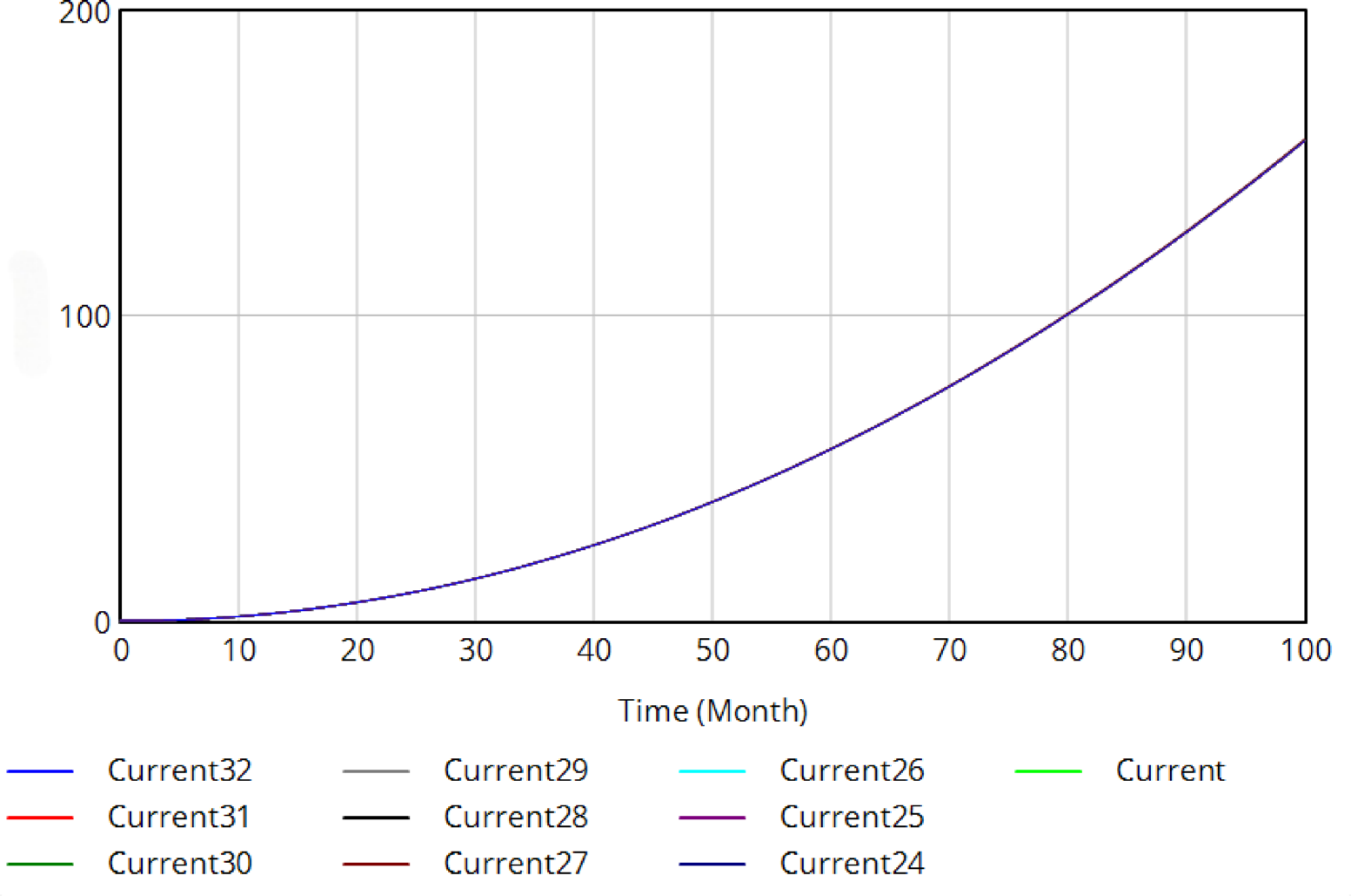
Changes in integrated resilience after sequentially changing factors of the emergency, rescue, and evacuation capabilities subsystem.

In this simulation process, the current represents resilience in emergency, rescue, and evacuation capabilities under original parameters. Similarly, the modified runs of current24-current32 represent emergency, rescue, and evacuation capabilities after individually increasing Fire station distribution (C24), Fire water supply (C25), Emergency infrastructure level (C26), Lifeline response capability (C27), Passage and exit distribution (C28), Passage and exit accessibility (C29), Emergency response teams (C30), Emergency communications and commands (C31), and Emergency medical facilities (C32) by 20%. Meanwhile, the initial value of each other index is unchanged. The changes in emergency, rescue, and evacuation capabilities are shown in Fig. 15, and the resilience changes of the gas system are shown in Fig. 16. Furthermore, the sensitivity analysis of emergency, rescue, and evacuation capabilities is shown in Table 13.

**Table 13:** Sensitivity analysis of emergency, rescue, and evacuation capabilities.

Status Number	Measures	Emergency, rescue, and evacuation capabilities	Growth rate	Gas system resilience	Growth rate
Current	Keep current status	0.0585	-	52.377	-
C24	Increase 20%	0.0599	2.39%	52.424	0.09%
C25	Increase 20%	0.0585	≈ 0%	52.377	≈ 0%
C26	Increase 20%	0.0585	≈ 0%	52.387	0.02%
C27	Increase 20%	0.0651	11.28%	52.596	0.42%
C28	Increase 20%	0.0614	4.96%	52.472	0.18%
C29	Increase 20%	0.0590	0.86%	52.394	0.03%
C30	Increase 20%	0.0585	≈ 0%	52.377	≈ 0%
C31	Increase 20%	0.0585	≈ 0%	52.377	≈ 0%
C32	Increase 20%	0.0585	≈ 0%	52.377	≈ 0%

#### Summary

Based on the resilience change amplitude induced by each factor, the sensitivity ranking is: C12 > C19 > C1 > C16 > C17 > C4 > C11 > C14 > C27 > C18 > C13 > C3 > C9 > C28 > C8 > C6 > C15 > C10 > C24 > C20 > C21 = C23 > C7 > C22 > C29 > C26 > C30 = C25 = C32 = C31 = C2 = C5. The sensitivity analysis of all factors is presented in Table 14.

Therefore, the data shows that proportion of high-pressure and Sub-HP pipelines (C12) is the most critical factor. A 20% improvement boosts system’s inherent resilience by 9.84%, Resilience of gas surrounding environment by 8.56%, and integrated resilience improvement by 7.39%. Similarly, building seismic resistance (C1) increases natural disaster resilience by 11.07% and integrated resilience by 1.85%. Flood control standards(C4) increase natural disaster resilience by 5.53% and integrated resilience by 0.94%. In addition, major hazard protection (C11) and pipeline corrosion factors assessment (C16) improve system’s inherent resilience by 1.60% and 3.08%, driving integrated resilience gains of 0.82% and 1.58% respectively. Lifeline response capability (C27) enhances emergency, rescue, and evacuation capabilities most significantly (11.28%), contributing to a 0.42% integrated resilience increase.

Based on sensitivity analysis, the following system-wide resilience improvement strategies are recommended for high-sensitivity indicators.

(1) Optimization of disaster defense and flood control planning.

Indicators C3 (Seismic secondary hazard prevention), C4 (flood control standards), and C6 (Flood non-engineering measures) exhibit strong interdependencies with the resilience of natural gas infrastructure against natural hazards. Secondary disasters e.g., fires and explosions triggered by seismic-induced gas leaks and flooding events can inflict severe damage on gas networks. System dynamics modeling reveals complex causal linkages between these factors and system stability. Current suboptimal performance and high sensitivity scores imply limited capacity to withstand disasters. To address C3, seismic-induced gas leak simulations should be intensified, emergency protocols refined, and response times accelerated through advanced training drills. Concurrently, investment in seismic retrofitting technologies for gas facilities must be prioritized. For C4, existing drainage standards require re-evaluation considering urban expansion and topographic shifts; drainage pipeline design capacities should be upgraded where necessary to enhance hydraulic efficiency. Regarding C6, a real-time flood monitoring and early-warning system must be established, with interagency data sharing to enable timely public alerts and preemptive mitigation.

(2) Protection and spatial adjustment of major hazard sources.

LNG stations and CNG stations are the critical nodes in gas supply chains. During initial planning phases, safety distances between refueling stations and residential or commercial zones should be expanded, and adjacent residential land converted to industrial use to minimize exposure to major hazard sources. Additionally, C11 (major hazard protection) and C12 (proportion of high-pressure and Sub-HP pipelines) directly influence system safety and operational stability. Uncontrolled major hazards could trigger catastrophic consequences, while suboptimal pipeline ratios compromise gas delivery efficiency and integrity. For C11, hazard identification, risk assessment, and monitoring frameworks must be strengthened through designated accountability, and advanced sensor deployment. Sector-specific emergency response plans should undergo regular validation via simulation exercises to bolster incident management capabilities. For C12, optimal pipeline ratios should be computed using professional network analysis software, aligned with urban gas demand patterns and development trajectories. Existing networks require phased retrofitting to reconfigure layouts, ensuring secure and efficient gas transmission.

(3) Optimization of Spatial Vulnerability in Urban Planning Layouts.

Indicators C18 (population density) and C19 (population-gas system spatial correlation) are critical to public safety, as gas incidents in crowded locations may cause mass casualties. Current deficiencies and high sensitivity stem from residential, commercial, and educational land uses collectively occupying 35–40% of total area, exacerbating vulnerability in high-density zones due to complex pipeline configurations. During planning optimization, land-use attributes should be reconfigured to reduce population concentration in high-risk areas. Specifically, residential area ratios must be decreased while industrial zones are expanded in high-risk areas. Furthermore, evacuation and rescue efficiency post-incident can be enhanced through strategic augmentation of access and emergency exits, improving spatial connectivity without compromising urban functionality.

**Table 14:** Trends in subsystems and integrated changes after changing initial values.

Status Number	Measures	Natural disaster risk prevention capability of gas system	Growth rate	Gas system’s inherent resilience	Growth rate	Resilience in gas surrounding environment	Growth rate	Emergency, rescue, and evacuation capabilities	Growth rate	Resilience of gas system	Growth rate
C0	Keep current status	0.271	-	0.813	-	0.444	-	0.0585	-	52.377	-
C1	Increase 20%	0.301	11.07%	0.813	≈ 0%	0.444	≈ 0%	0.0585	≈ 0%	53.345	1.85%
C2	Increase 20%	0.271	≈ 0%	0.813	≈ 0%	0.444	≈ 0%	0.0585	≈ 0%	52.377	≈ 0%
C3	Increase 20%	0.276	1.85%	0.813	≈ 0%	0.444	≈ 0%	0.0585	≈ 0%	52.530	0.29%
C4	Increase 20%	0.286	5.53%	0.813	≈ 0%	0.444	≈ 0%	0.0585	≈ 0%	52.867	0.94%
C5	Increase 20%	0.272	0.37%	0.813	≈ 0%	0.444	≈ 0%	0.0585	≈ 0%	52.388	≈ 0%
C6	Increase 20%	0.273	0.74%	0.813	≈ 0%	0.444	≈ 0%	0.0585	≈ 0%	52.441	0.12%
C7	Increase 20%	0.272	0.37%	0.813	≈ 0%	0.444	≈ 0%	0.0586	0.17%	52.403	0.05%
C8	Increase 20%	0.273	0.74%	0.813	≈ 0%	0.444	≈ 0%	0.0587	0.34%	52.452	0.14%
C9	Increase 20%	0.271	≈ 0%	0.816	0.37%	0.444	≈ 0%	0.0585	≈ 0%	52.476	0.19%
C10	Increase 20%	0.271	≈ 0%	0.815	0.25%	0.444	≈ 0%	0.0585	≈ 0%	52.428	0.10%
C11	Increase 20%	0.271	≈ 0%	0.826	1.60%	0.444	≈ 0%	0.0585	≈ 0%	52.808	0.82%
C12	Increase 20%	0.271	≈ 0%	0.893	9.84%	0.482	8.56%	0.0585	≈ 0%	56.247	7.39%
C13	Increase 20%	0.271	≈ 0%	0.819	0.74%	0.444	≈ 0%	0.0585	≈ 0%	52.563	0.36%
C14	Increase 20%	0.271	≈ 0%	0.825	1.48%	0.444	≈ 0%	0.0585	≈ 0%	52.766	0.74%
C15	Increase 20%	0.271	≈ 0%	0.815	0.25%	0.444	≈ 0%	0.0585	≈ 0%	52.437	0.11%
C16	Increase 20%	0.271	≈ 0%	0.838	3.08%	0.444	≈ 0%	0.0585	≈ 0%	53.205	1.58%
C17	Increase 20%	0.271	≈ 0%	0.834	2.58%	0.444	≈ 0%	0.0585	≈ 0%	53.073	1.33%
C18	Increase 20%	0.271	≈ 0%	0.813	≈ 0%	0.450	8.56%	0.0585	≈ 0%	52.572	0.37%
C19	Increase 20%	0.271	≈ 0%	0.813	≈ 0%	0.485	1.35%	0.0585	≈ 0%	53.728	2.58%
C20	Increase 20%	0.271	≈ 0%	0.813	≈ 0%	0.446	9.23%	0.0585	≈ 0%	52.419	0.08%
C21	Increase 20%	0.271	≈ 0%	0.813	≈ 0%	0.446	0.45%	0.0585	≈ 0%	52.415	0.07%
C22	Increase 20%	0.272	≈ 0%	0.813	≈ 0%	0.445	0.45%	0.0585	≈ 0%	52.399	0.04%
C23	Increase 20%	0.271	≈ 0%	0.813	≈ 0%	0.446	0.23%	0.0585	≈ 0%	52.416	0.07%
C24	Increase 20%	0.271	≈ 0%	0.813	≈ 0%	0.444	≈ 0%	0.0599	2.39%	52.424	0.09%
C25	Increase 20%	0.271	≈ 0%	0.813	≈ 0%	0.444	≈ 0%	0.0585	≈ 0%	52.377	≈ 0%
C26	Increase 20%	0.272	0.37%	0.813	≈ 0%	0.444	≈ 0%	0.0585	≈ 0%	52.387	0.02%
C27	Increase 20%	0.271	≈ 0%	0.813	≈ 0%	0.444	≈ 0%	0.0651	11.28%	52.596	0.42%
C28	Increase 20%	0.271	≈ 0%	0.813	≈ 0%	0.444	≈ 0%	0.0614	4.96%	52.472	0.18%
C29	Increase 20%	0.271	≈ 0%	0.813	≈ 0%	0.444	≈ 0%	0.0590	0.86%	52.394	0.03%
C30	Increase 20%	0.271	≈ 0%	0.813	≈ 0%	0.444	≈ 0%	0.0585	≈ 0%	52.377	≈ 0%
C31	Increase 20%	0.271	≈ 0%	0.813	≈ 0%	0.444	≈ 0%	0.0585	≈ 0%	52.377	≈ 0%
C32	Increase 20%	0.271	≈ 0%	0.813	≈ 0%	0.444	≈ 0%	0.0585	≈ 0%	52.377	≈ 0%

## Discussion

This study demonstrates that system’s inherent resilience (A2) is the dominant contributor to urban gas infrastructure performance, with the proportion of high-pressure pipelines (C12) identified as the most sensitive indicator. A 20% improvement in C12 yields a 7.39% increase in integrated system resilience. This finding aligns with previous research identifying pipeline characteristics as critical to gas system safety, but extends it by quantifying the marginal benefits of planning-stage interventions over operational measures.

Conversely, the consistently low sensitivity of emergency, rescue, and evacuation indicators (A4) requires equally careful interpretation. This result may reflect structural features of the model, such as the distance of these factors from primary causal pathways, rather than real-world insignificance. Planners should therefore interpret this finding cautiously: it suggests that under normal operating conditions and moderate disruptions, foundational design choices matter most for resilience. However, this does not imply that emergency capabilities are unimportant, particularly for extreme events where rapid response can be decisive. Comprehensive resilience strategies must therefore integrate both robust foundational design and adequate emergency response capacity.

From a practical perspective, indicators producing integrated resilience changes below 1% should be interpreted as having negligible practical significance within this framework. Planners may safely deprioritize these factors unless other considerations, such as regulatory requirements, stakeholder concerns, or cost-effectiveness, mandate attention. This provides a clear threshold for resource allocation, focusing investments on factors that meaningfully influence resilience outcomes.

From an operational perspective, the planning-phase approach presented here aligns with emerging resilience-by-design frameworks being developed in other infrastructure contexts. The framework can be integrated into planning support systems. The indicator scores and sensitivity rankings can be visualized within GIS-based platforms to identify risk hotspots and evaluate alternative land-use scenarios. Coupled with digital twins, the model could provide dynamic feedback as planning parameters evolve, enabling real-time assessment of resilience implications for utility planning and investment decisions.

Several limitations should be acknowledged. First, the reliance on expert scoring introduces potential subjectivity, though this was mitigated through diverse expert selection (from industry, government, and academia), a Delphi-style feedback process, and inter-rater reliability testing (Kendall’s W = 0.72, *p* < 0.01). Second, the case study’s specificity to a Chinese new district may limit generalizability to contexts with different regulatory or hazard profiles. Third, the model does not explicitly incorporate climate change scenarios, which could alter hazard intensities over longer planning horizons. Fourth, the use of linear weighted summation, while transparent, may not fully capture nonlinear interactions among risk factors; in reality, synergistic effects could amplify the impacts suggested by linear combination. Fifth, the single-factor sensitivity analysis may not reflect real-world interventions that affect multiple factors simultaneously. Although preliminary exploration of selected two-factor combinations (e.g., C12 + C19) showed approximately additive effects, more comprehensive analysis of synergistic interactions across the full indicator set remains an important direction for future research. Finally, the lack of quantitative validation against historical data is acknowledged, as this is a common constraint in planning-stage research where no comparable resilience measurements exist for newly planned infrastructure.

Despite these limitations, the robustness tests provide confidence in the main findings. Sensitivity rankings remained consistent across alternative simulation horizons (50, 100, and 200 months), disturbance levels (10%, 20%, and 30%), and model specifications (alternative weight sets and aggregation functions). This consistency supports the conclusion that planning-phase interventions targeting high-sensitivity indicators offer substantially greater resilience dividends than post-construction emergency measures.

## Conclusions

This study established a system dynamics-based framework to identify and prioritize risk factors for enhancing urban gas infrastructure resilience during the planning stage. Three main contributions emerge from this work.

First, a structured framework was developed, comprising a three-tier indicator system of 32 factors across four resilience dimensions, integrated with AHP weighting and SD modeling. This framework enables systematic sensitivity analysis for new urban districts where empirical data are limited, providing a replicable methodology for planning-stage resilience assessment.

Second, the key findings identify system’s inherent resilience (A2) as the dominant contributor to integrated performance, with the proportion of high-pressure pipelines (C12) as the most sensitive indicator where a 20% improvement yields a 7.39% resilience gain. Population-gas spatial coupling (C19) and building seismic resistance (C1) follow as high-impact intervention points. These quantifiable results provide empirical evidence for prioritizing planning investments.

Third, the analysis yields practical guidance for urban planners and infrastructure managers: (1) optimize disaster defense through seismic retrofitting and flood standard re-evaluation; (2) adjust major hazard siting by expanding safety distances between LNG/CNG facilities and residential areas; and (3) reconfigure land use to reduce population concentration in high-risk zones while enhancing evacuation pathways.

By demonstrating that planning-phase interventions offer substantially greater resilience dividends than post-construction emergency measures, this framework challenges conventional priorities and provides evidence-based support for preemptive risk mitigation. The approach establishes that resilience should be “baked in” during planning rather than “bolted on” after construction.

Future research should extend this framework by incorporating climate change projections, exploring nonlinear and synergistic interactions among risk factors, and validating the approach across diverse urban contexts with varying regulatory and hazard profiles.

## Data Availability

Some or all data, models, or code that support the findings of this study are available from the corresponding author upon reasonable request.
